# The architecture of the *Schizosaccharomyces pombe* CCR4-NOT complex

**DOI:** 10.1038/ncomms10433

**Published:** 2016-01-25

**Authors:** Marta Ukleja, Jorge Cuellar, Aleksandra Siwaszek, Joanna M. Kasprzak, Mariusz Czarnocki-Cieciura, Janusz M. Bujnicki, Andrzej Dziembowski, Jose M. Valpuesta

**Affiliations:** 1 Institute of Biochemistry and Biophysics, Polish Academy of Sciences, 02-106 Warsaw, Poland; 2 Department of Genetics and Biotechnology, Faculty of Biology, University of Warsaw, 02-106 Warsaw, Poland; 3 Department of Structure of Macromolecules, Centro Nacional de Biotecnología (CNB-CSIC), Darwin 3, 28049 Madrid, Spain; 4 Laboratory of Bioinformatics and Protein Engineering, International Institute of Molecular and Cell Biology in Warsaw, ul. Ks. Trojdena 4, PL-02-109 Warsaw, Poland; 5 Bioinformatics Laboratory, Institute of Molecular Biology and Biotechnology, Faculty of Biology, Adam Mickiewicz University, ul. Umultowska 89, PL-61-614 Poznan, Poland

## Abstract

CCR4-NOT is a large protein complex present both in cytoplasm and the nucleus of eukaryotic cells. Although it is involved in a variety of distinct processes related to expression of genetic information such as poly(A) tail shortening, transcription regulation, nuclear export and protein degradation, there is only fragmentary information available on some of its nine subunits. Here we show a comprehensive structural characterization of the native CCR4-NOT complex from *Schizosaccharomyces pombe*. Our cryo-EM 3D reconstruction of the complex, combined with techniques such as immunomicroscopy, RNA-nanogold labelling, docking of the available high-resolution structures and models of different subunits and domains, allow us to propose its full molecular architecture. We locate all functionally defined domains endowed with deadenylating and ubiquitinating activities, the nucleus-specific RNA-interacting subunit Mmi1, as well as surfaces responsible for protein–protein interactions. This information provides insight into cooperation of the different CCR4-NOT complex functions.

## Abstract (web summary)

CCR4-NOT is a protein complex involved in a variety of important genetic processes. Here, the authors report the mid-resolution structure of this complex, and model the positions and contacts between the subunits, providing structural support for the previously reported functions of the complex.

## Graphical abstract

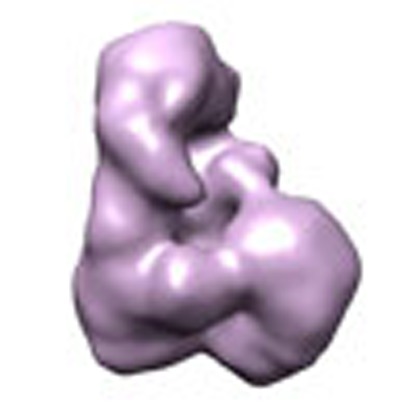

The CCR4-NOT complex is a large macromolecular assembly involved in many different aspects of mRNA processing in the nucleus and in cytoplasm, including chromatin modification, transcription elongation, mRNA degradation, miRNA gene silencing, RNA nuclear surveillance and nuclear export^1^,^2^. Its vital role in gene expression is reflected in its high conservation across eukaryotes. The CCR4-NOT complex was initially characterized as a global (positive and negative) regulator of transcription^3^, but its functions are now known to be much broader. CCR4-NOT is a cytoplasmic deadenylase that determines the half-life of mRNA molecules, and is responsible for the distributive shortening of the poly(A) tail before mRNA is degraded by the 3′-5′ or 5′-3′ mRNA decay pathways. Due to its ubiquitinating activity, it also regulates protein solubility and co-translational quality control^4^,^5^.

The CCR4-NOT complex consists of eight main subunits. Not1 (negative on TATA 1) is the largest of these (237 kDa) and forms a scaffold for the complex assembly. Ccr4 (76 kDa) and Caf1 (usually termed Pop2 in *Schizosaccharomyces pombe*; 37.5 kDa) act as deadenylases^6^. Not4 (54 kDa) is responsible for the second enzyme activity, ubiquitination^4^. The remaining subunits, Not2, Not3 and Not5 (34, 73 and 66 kDa, respectively), form the so called Not module, with no clear function yet assigned^7^. Caf40 (Ccr4-associated factor 40; 32 kDa) was recently reported to have a role in RNA degradation as part of the miRNA machinery^8^,^9^. Composition of the complex nonetheless varies between species. For instance, *Saccharomyces cerevisiae* CCR4-NOT has an additional subunit, Caf130, and its probable functional analogue is reported to be part of the *Drosophila* (Not10) (ref. 10) and human (Cnot10) (ref. 11) CCR4-NOT complexes. In metazoans, Cnot10 interacts with Cnot11 and Cnot1, forming a separate module of the complex^10^,^11^. In *S. pombe*, a nuclear form of the CCR4-NOT complex interacts with the RNA-binding protein Mmi1 (a 54 kDa YTH family RNA-binding protein) (A. Siwaszek and A. Dziembowski, in preparation), responsible for degradation and silencing of meiotic transcripts during vegetative growth, which also recruits the complex to its target mRNA^12^.

As a multifunctional assembly, CCR4-NOT interacts with a variety of cofactors that help recruit the complex to its mRNA targets. A CCR4-NOT-mediated mechanism to induce degradation and inhibit translation of the miRNA targets was recently proposed^8^,^9^. Cnot9 (the human homologue of yeast Caf40) binds to the central region of human Not1 (Cnot1) and interacts with GW182 (TNRC6), one of the main effectors of the miRNA degradation pathway. The entire CCR4-NOT complex is thus recruited to the mRNA directed to degradation. Moreover, the Cnot1 MIF4G domain interacts with the translation repressor and decapping activator DDX6 (refs 8, 9). CCR4-NOT uses a similar mechanism to interact with different RNA-binding proteins that recruit the complex to the target mRNA; the messenger RNA is degraded in a CCR4-NOT-mediated, deadenylation-dependent manner. The best-characterized of these binding partners are tristetraprolin (TTP)^13^, Nanos1 (ref. 14) and Tob/BTG^15^.

CCR4-NOT was recently defined as a molecular linker between the transcription and translation processes^16^,^17^. Initial studies showed that CCR4-NOT complex subunits crosslink to genes during transcription^18^, interact with RNA Pol II, and regulate transcription elongation^19^. A connection was proposed between CCR4-NOT subunit Not5 and the Rbp4/7 module of RNA Pol II^16^,^17^. The physical interaction has now been demonstrated, and seems to be crucial for Rbp4 cytoplasmic function, its association with mRNA and regulation of mRNA degradation and translation. The CCR4-NOT was also implicated in rescuing arrested RNA Pol II elongation complex in a Rbp4/7 heterodimer-dependent manner^16^. Not5 contributes to the cytoplasmic assembly of RNA Pol II^16^. Finally, the Not4 ubiquitinating activity widens the spectrum of CCR4-NOT functions in the cell, and includes general and co-translational quality control^4^,^20^,^21^ and proteasome assembly.

The cooperation mechanism among all CCR4-NOT functions remains largely unknown, due in part to the lack of structural information for the whole complex, although data for some of its components are available; most of the currently known structural data have been comprehensively reviewed by Xu *et al*.^22^ High-resolution structures have been reported for several CCR4-NOT subunits and/or domains, including the nuclease domain of Ccr4 (ref. 23), and the Not4 RING^24^ and RNA recognition motif domains. Structures of functional assemblies have been determined, such as that of the nuclease module consisting of Ccr4, Caf1 and a small Not1 fragment that incorporates the nucleases into the complex^25^, the Not module (Not2-Not5-Not1) (ref. 7), or structures of complete subunits such as Pop2/Caf1refs 26,27 and Caf40 (ref. 28). The recent work of Bhaskar *et al*.^29^ presented the crystal structure of the yeast Not4 C-terminal fragment bound to the Not1C-terminal domain, together with the Not4 N-terminal RING domain that interacts with its functional partner, Ubc4. The lack of a high-resolution structure for the entire CCR4-NOT complex is due to the current inability to obtain crystals, mainly because of the large size of the complex, the flexibility of some of its components, and the presence of unstructured regions.

Electron microscopy (EM) offers an alternative approach to solve the structure of the complex macromolecular assemblies, but the only structural data for the whole assembly, thus far, is derived from a low-resolution (30 Å) structure of the *S. cerevisiae* complex^30^. Combination of medium/low-resolution 3D information from EM of a macromolecular complex and the docking of the high-resolution structures of components of the complex is nonetheless a powerful tool that can be used to generate a pseudo-atomic model of a complex under study^31^,^32^.

In our analysis, the CCR4-NOT complex from *S. pombe* was expressed endogenously, purified, and its structure generated using cryo-EM and single-particle 3D reconstruction. The combination of immunomicroscopy and RNA-nanogold labelling techniques, coupled with the pseudo-atomic model of all components of the complex based on available high-resolution structures of subunits and domains, allows us to provide the first full molecular architecture of CCR4-NOT.

## Results

### Purification of CCR4-NOT and *in vitro* enzymatic activity

**Figure 1:**
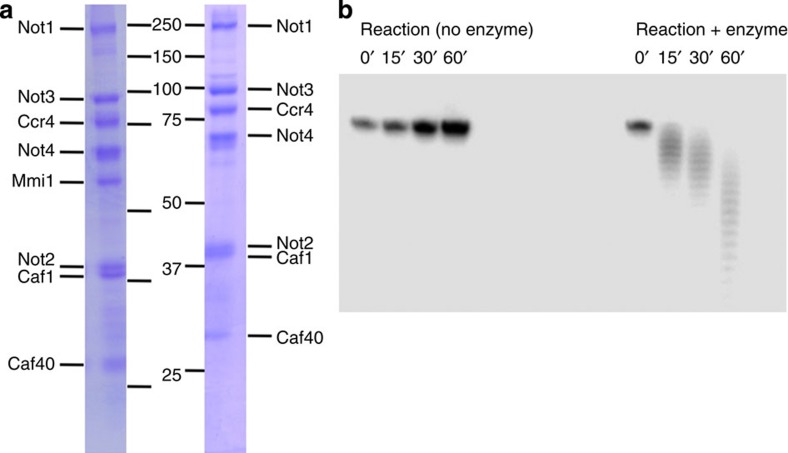
Purification of the active CCR4-NOT complex from fission yeast *S. pombe.* (**a**) SDS-PAGE analysis of the purified CCR4-NOT complex (left) and the CCR4-NOT complex lacking Mmi1 (Mmi1ΔCCR4-NOT complex; right). (**b**) *In vitro* deadenylation assay. CCR4-NOT deadenylation was assessed using polyadenylated RNA substrate (34 nucleotides) and the purified CCR4-NOT complex. Samples were collected at 0, 15, 30 and 60 min. The control reaction had no added protein.

To analyze the structure of the *S. pombe* CCR4-NOT complex, we devised a purification protocol that combines Protein A tag and IgG affinity chromatography, followed by glycerol gradient centrifugation (Fig. 1a). The purification conditions allowed maintenance of the complex in a near-native state throughout the procedure, and high salt conditions were used to avoid non-specific protein–protein and/or protein–RNA interactions. Mass spectrometry analysis confirmed the presence of all canonical subunits described in other model systems, (Not1-4, Ccr4, Caf1, Caf40) and a *S. pombe*-specific subunit, Mmi1. Some degradation was also visible, in particular of Not1, Not4 and Caf40, together with some minor contaminants.

To test the function of the purified CCR4-NOT complex, we assayed one of its enzymatic activities, the deadenylation of polyadenylated mRNA substrates by the two active exoribonuclease subunits, Ccr4 and Caf1. We observed gradual degradation of the polyadenylated RNA substrate (Fig. 1b), characteristic of the distributive enzymes. The assay confirmed conservation of enzyme activity in the purified complex, which indicates that the complex retains its native state, essential for reliable structural analysis.

### Structural characterization of CCR4-NOT by cryo-EM

**Figure 2:**
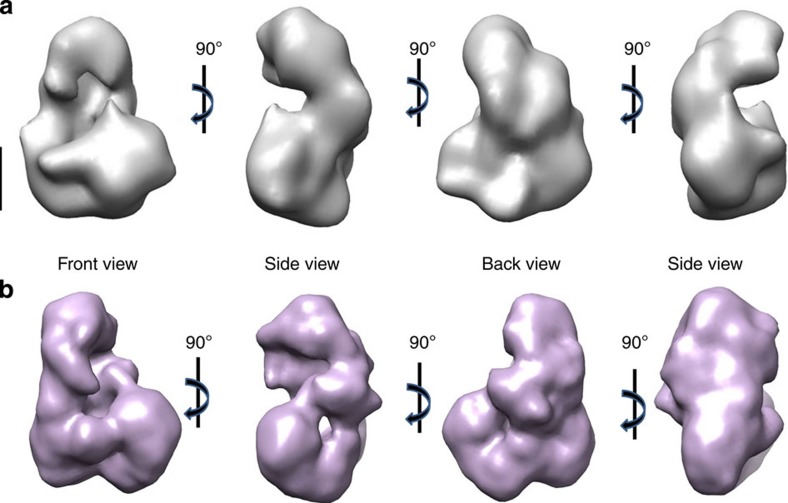
Architecture of the CCR4-NOT complex from *S. pombe*. (**a**) Four orthogonal views of the CCR4-NOT 3D reconstruction by negative staining EM (23 Å resolution). (**b**) The views as in **a** of the complex by cryo-EM (20 Å resolution). Scale bar, 50 Å.

The glycerol gradient fraction with the highest CCR4-NOT concentration and purity was analyzed by EM, initially using negative staining. A total of 10,303 particles were selected and classified using ML2D and CL2D methods^33^,^34^ (Supplementary Fig. 1a,b). The classes selected were used for 3D reconstruction (see Online Methods). The final structure obtained has a curved L-like shape, with one arm ∼150 Å long and another slightly shorter (∼140 Å), and a cavity between these two masses (Fig. 2a). The overall shape of the complex is similar to that obtained for *S. cerevisiae*^30^, which indicates a common structure for these complexes.

To generate a more faithful, detailed structure of CCR4-NOT, we analyzed the sample using cryo-EM. The standard purification procedure imposed certain limitations such as low concentration as well as glycerol in the sample that could not be removed completely, which reduced contrast in the cryo-EM analysis. To stabilize the complex and increase sample homogeneity, we applied the GraFix method, a gradient technique for purifying and stabilizing macromolecular complexes at low glutaraldehyde concentration, particularly suitable for EM^35^. The stabilized sample was concentrated by centrifugation using Amicon centrifuge membranes and subsequently vitrified (Supplementary Fig. 1c). A total of 60,480 particles were selected manually and classified using a CL2D procedure^33^. The 2D classes generated showed the typical orientations observed in negative staining EM analysis (Supplementary Fig. 1d and Fig. 2a). A subset of 20,500 best-quality particles was selected for 3D reconstruction (for the protocol, see Online Methods and Supplementary Figs 2 and 3). The volume generated (20 Å resolution; Fig. 2b) is similar in structure and dimensions to that obtained using a heavy metal salt, except that it shows more detailed features and a cavity in the centre of the shorter arm.

### Mapping of Mmi1 into the CCR4-NOT complex

In addition to the canonical subunits described for the complex in other organisms^11^,^36^,^37^ the *S. pombe* CCR4-NOT complex contains the Mmi1 nuclear RNA-binding protein. This protein is responsible for removing meiosis-specific transcripts during vegetative growth^38^. It recognizes the hexanucleotide motif U(U/C)AAAC, called the determinant of selective removal (DSR), which is located in the 3′-untranslated region of meiosis-specific mRNA^38^. After binding, the transcript is degraded. Mmi1 is a stable component of the *S. pombe* CCR4-NOT complex, although the biological relevance of this interaction remains unclear.

**Figure 3:**
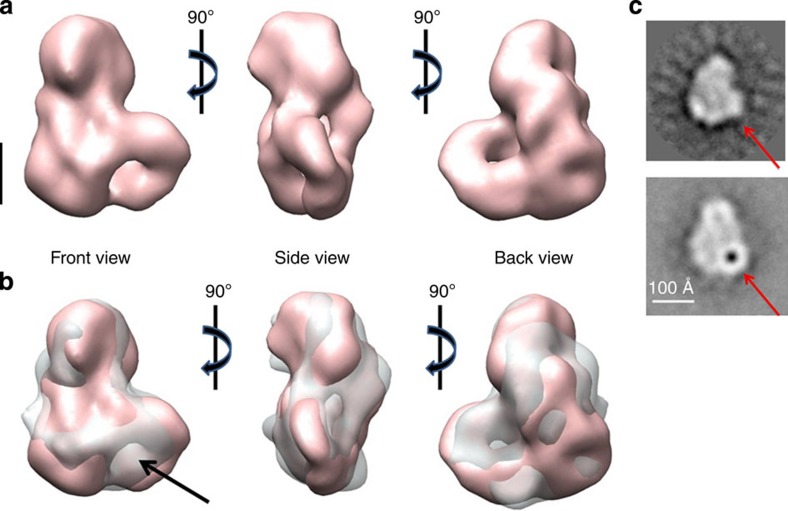
Location of the Mmi1 subunit in the CCR4-NOT complex. (**a**) Three orthogonal views of the 3D reconstruction of the Mmi1ΔCCR4-NOT complex (20 Å resolution). (**b**) Superimposition of the 3D reconstruction of Mmi1ΔCCR4-NOT (pink, solid volume) on the CCR4-NOT complex (transparent, grey volume). Comparison of the two reconstructions shows an additional mass in the CCR4-NOT complex (black arrow). (**c**) 2D classes representing the front view of the CCR4-NOT complex. Top, 2D class of the complex used as a reference for particle alignment. Bottom, 2D class of the complex incubated with the nanogold-labelled Mmi1-binding RNA. Black dot, a nanogold particle visualized by EM. Red arrow, position of the Mmi1 subunit. Scale bar, 50 Å.

To locate the position of Mmi1 in the 3D reconstruction of the complex, we carried out EM analysis of a CCR4-NOT complex lacking Mmi1 (ΔMmi1CCR4-NOT). The *Mmi1* gene is essential, which precludes simple knockout strategies; therefore we used a subtraction strategy to purify the CCR4-NOT complex without Mmi1. We constructed a *S. pombe* strain in which the Not2 subunit was fused to protein A and TEV protease cleavage sites, as well as a *Mmi1* gene fused to that of protein A tag lacking the TEV protease cleavage site. This strategy allowed purification of CCR4-NOT complex lacking Mmi1, since CCR4-NOT is released after TEV cleavage, while Mmi1 remains attached to resin beads. The purified complex was analyzed by SDS-polyacrylamide gel electrophoresis (PAGE), and the lack of Mmi1 protein was confirmed by the absence of a band migrating at ∼55 kDa (Fig. 1a). Negatively stained micrographs of the complex showed a homogenous population of particles. A total of 10,331 particles were selected, aligned and classified. The 2D classification showed a set of different views comparable to the classes obtained for the entire CCR4-NOT complex. The 3D reconstruction procedure rendered a volume very similar to that of wild-type CCR4-NOT (Fig. 3a,b). There was nonetheless a clear difference between the two volumes; a small channel in the shorter mass of the ΔMmi1CCR4-NOT complex was linked to the absence of Mmi1, and was observed clearly when the two volumes were superimposed (Fig. 3b). To confirm Mmi1 localization, we labelled CCT4-NOT with the RNA oligonucleotide consisting of four tandem DSR motifs fused to Nanogold (termed oligo-DSR). Negative staining EM analysis of the oligo-DSR-Nanogold-bound complex showed considerable heterogeneity, probably due to the presence of RNA. A total of 8,500 Nanogold-bound particles (visible as black dots, due to high electron scattering of the Nanogold particles) were selected manually and classified by CL2D and ML2D. To determine Mmi1 location based on the position of Nanogold in the particles, we selected the characteristic L-shape view from the 2D classes of the CCR4-NOT complex, which we used as a reference for alignment of the Nanogold-bound particles. The 2D average obtained shows the position of the Nanogold and thus, indirectly, that of Mmi1 (Fig. 3c) in the centre of the shorter arm, in accordance with the result of the 3D reconstruction of ΔMmi1CCR4-NOT.

### Localization of the CCR4-NOT subunits by immunomicroscopy

We then sought to locate the CCR4-NOT complex subunits. Since the low-resolution 3D model of the entire complex does not allow their direct localization, we used immuno-EM to locate each subunit within the 3D reconstruction of CCR4-NOT. The complex was purified from several yeast strains (Supplementary Table 1), in which Not2 was fused to an affinity tag, and green fluorescent protein (GFP) to the C terminus of the distinct target subunits. The growth rate of the GFP-fusion *S. pombe* strains was comparable to that of wild type. We confirmed the correct translational fusion and expression of modified complex subunits using western blot analysis with an anti-GFP antibody. After the first purification step, the complexes were incubated with the anti-GFP monoclonal antibody or Fab fragment and immunocomplexes were loaded onto a glycerol gradient to separate the immunocomplex from unbound antibody and impurities. Glycerol gradient fractions were analyzed by SDS-PAGE. All eight CCR4-NOT complex subunits were found in the middle fractions (Supplementary Fig. 4), with two additional bands that migrated at ∼50 and ∼25 kDa, corresponding to antibody heavy and light chains. Immunocomplex migration within the density gradient was comparable to that of wild-type CCR4-NOT, which confirms correct assembly despite the presence of the GFP tag and bound antibody.

**Figure 4:**
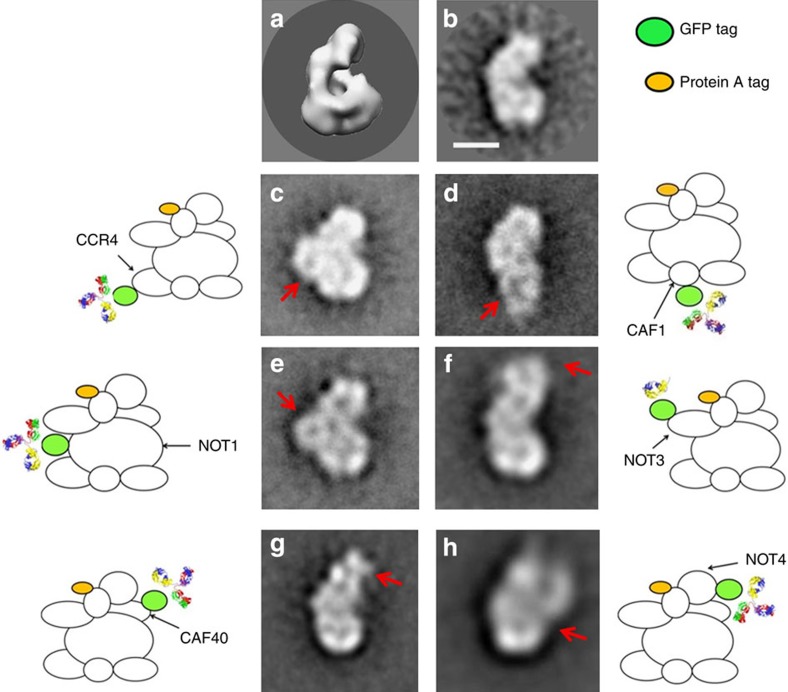
Immunolocalization of the CCR4-NOT complex subunits. (**a**) Front view of the CCR4-NOT 3D reconstruction. (**b**) 2D class average used as a reference for alignment of the selected particles with bound antibody. (**c**–**h**) The same views of the immunocomplexes formed by CCR4-NOT subunits and antibody. The red arrow indicates the additional density in each immunocomplex, which corresponds to the antibody or Fab fragment bound.

The glycerol gradient fraction containing the immunocomplexes was analyzed by negative staining EM. For each immunocomplex, >10,000 particles were selected manually (see Supplementary Table 2) and subjected to KerDenSom^39^, which classifies prealigned particles by focusing mainly on a specific region of the image, defined by a predesigned mask. Particles from the selected classes were extracted and aligned using a 2D average of the CCR4-NOT representing the typical L-shaped front view (Fig. 4a,b) and classified by KerDenSom. Application of specific masks that focused on different areas of the 2D image identified protrusions in different parts when compared with the 2D average of the complex without antibody (Fig. 4b). Using this protocol, we located nucleases Ccr4 and Caf1 in the central part of the structure and in the shorter arm of the complex, respectively (Fig. 4c,d). They are near one another, which coincides with the high-resolution structure of the nuclease module^25^. The C terminus of the large scaffolding Not1 was found in the middle part of the front view (Fig. 4e), in a position similar to that of Ccr4, which suggests that the C-terminal ends of both subunits are located in a similar region of CCR4-NOT. In the case of Not3, the antibody binds at the top of the longer arm (Fig. 4f), opposite Caf1 and the rest of the nuclease module. As shown in the atomic structure, Not5 (a Not3 orthologue^40^) interacts with Not2 and with the Not1 C terminus^7^, which suggests that Not2 and the whole Not module are located in the long arm of the L-shaped complex. Antibody localization of Caf40 in CCR4-NOT (Fig. 4g) indicates the upper region of the long arm, near Not2 and Not3. Finally, Not4 was found in the central part of the structure, opposite the Not1 and Ccr4 C termini (Fig. 4h).

### Structural characterization of the Not2-Not5 heterodimer

**Figure 5:**
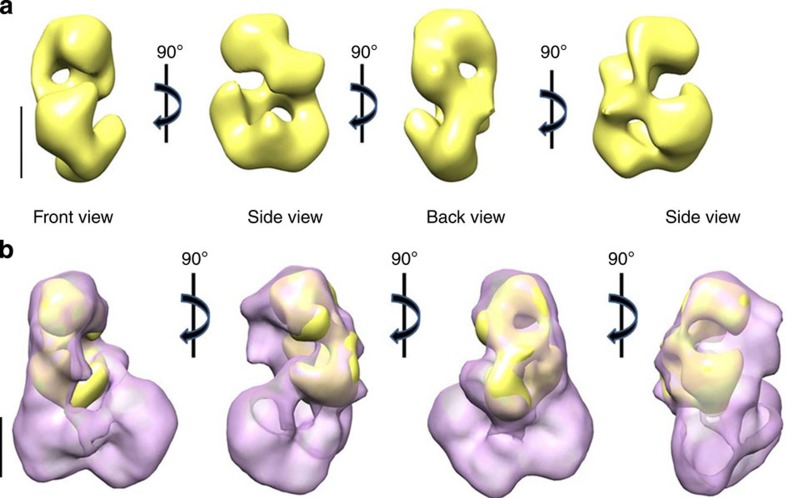
3D reconstruction of the yeast Not2-Not5 heterodimer. (**a**) Four orthogonal views of the 3D reconstruction of the Not2-Not5 heterodimer (16 Å resolution). (**b**) Manual docking of the 3D reconstruction of Not2-Not5 (yellow) within the CCR4-NOT cryo-EM 3D reconstruction (purple). Scale bar, 50 Å.

In *S. pombe*, man, *Drosophila* and *Trypanosoma*, a single gene encodes Not3 protein. It is an orthologue of *S. cerevisiae* Not3 and Not5 genes, products of gene duplication. The proteins share a homologous C-terminal domain, the NOT-box, whereas their N termini are very different^40^. The baker's yeast CCR4-NOT Not2 (23 kDa) and Not5 (73 kDa) subunits (Not2-Not3in *S. pombe*, man, *Drosophila* and *Trypanosoma*) form a stable heterodimer (Not2-Not5 in *S. cerevisiae*). The *S. cerevisiae* dimer was overexpressed, purified in *Escherichia coli* and complex formation was confirmed by size-exclusion chromatography. Negative staining EM analysis of the Not2-Not5 dimer showed a population of particles homogenous in size and shape (Supplementary Fig. 5a). A total of 20,055 particles were then selected and classified by CL2D and ML2D methods. The 2D classes, which confirmed sample homogeneity and showed general features of the complex (Supplementary Fig. 5b), were used for 3D reconstruction. The 3D model showed two connected masses, one ∼110 Å long and one of ∼70 Å (Fig. 5a). The central part of the volume encompasses a spacious cavity, and this space could be occupied by the C-terminal part of Not1 in accordance with the crystal structure of the yeast Not1-Not2-Not5-module^7^.

The immunolocalization experiments indicated the position of the *S. pombe* Not3 subunit (Fig. 4f), which is an orthologue of *S. cerevisiae* Not5, a component of the reconstructed Not2-Not5 heterodimer. We used this information to locate the heterodimer in the cryo-EM 3D reconstruction of CCR4-NOT, which was further improved by manual fitting using Chimera software (Fig. 5b). The suggested Not2-Not5 position occupies most of the CCR4-NOT long arm, leaving the shorter arm for the nuclease module.

### Pseudo-atomic model building of the CCR4-NOT complex

**Figure 6:**
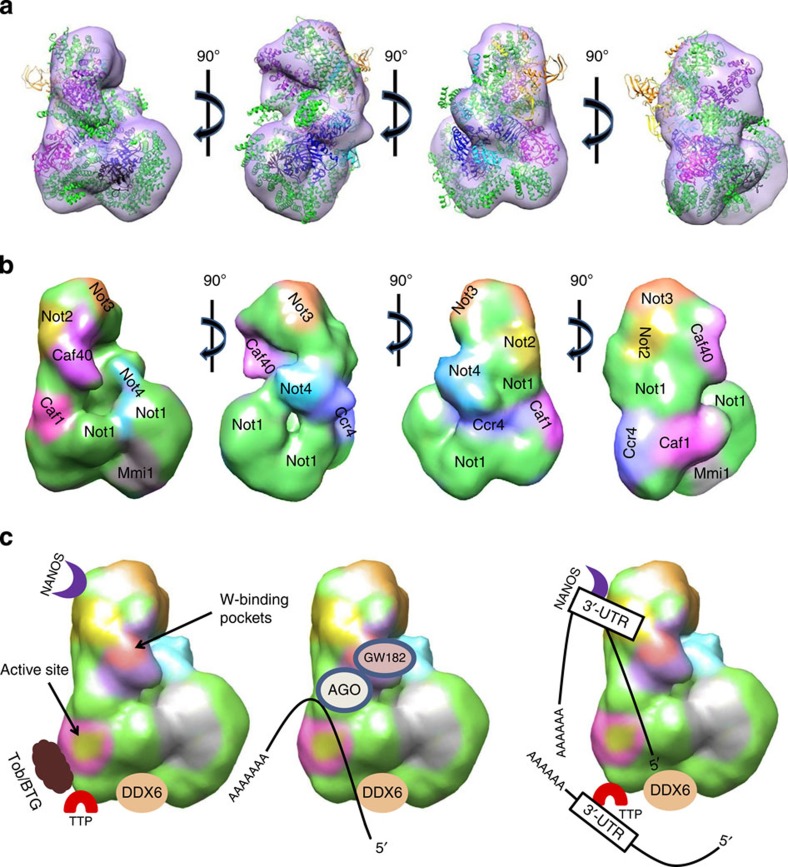
Functional implications of CCR4-NOT subunit organization. (**a**) Pseudo-atomic model generated after model building. (highly disordered regions were removed from the model, but their location for each subunit is suggested by the color codes in **b** and **c** (**b**) Map of the CCR4-NOT complex subunits. Expanded coloured regions of Not2, Not3, Not4 and Mmi1 represent the disordered fragments. (**c**) Left, binding sites of the known CCR4-NOT-interacting partners Tob/BTG (which binds to Caf1) and Nanos, TTP and DDX6 (which bind to different parts of Not1). The Caf1 deadenylase active site is indicated in yellow and Caf40 W-binding pockets in pink. Centre, proposed mechanism of miRNA substrate degradation. Caf40 recruits RITS complex through GW182 binding; the mRNA 5′ end interacts with DDX6, which enhances its decapping activity, while the 3′ end is deadenylated by Caf1. Right, mechanism for degradation of mRNA recruited by the mRNA-binding proteins Nanos and TTP. These proteins bind to the 3′-UTR of mRNA, which they recruit to the CCR4-NOT complex.

To obtain a structural model of the entire CCR4-NOT complex, we applied a computational modelling approach. First, we built homology models of all the CCR4-NOT components based on the *S. pombe* sequences and available high-resolution structures of orthologous proteins (Supplementary Fig. 6) and domains. We then docked all experimentally determined or modelled structures of all components into the electron density map of the complex using Situs^41^ and ADP_EM^42^ software. This docking confirmed the positions of Ccr4-Caf1-Not1, Caf40-Not1, Not2-Not3-Not1 and Not4-Not1 modules in the volume identified in the immunolabeling experiments, but did not allow us to build a model of the entire complex. We therefore applied a hybrid modelling approach using PyRy3D software (http://genesilico.pl/pyry3d/), which allows simultaneous docking of structures into the density map using spatial restraints and by modelling disordered and flexible regions during complex assembly. Spatial restraints of the complex subunits were derived from the EM analysis, supported by information on binary interactions between CCR4-NOT components^7^,^9^,^25^ (see the Methods section for details). Finally, we provided Situs-generated positions of Ccr4-Caf1-Not1, Caf40-Not1, Not2-Not3-Not1 and Not4-Not1 modules as starting positions for PyRy3D, and subsequently applied Monte Carlo simulations to optimize these positions in the EM map and to determine orientations of the remaining proteins (Mmi1, part of Not1 residues 1,500–1,850). We clustered the 100 best-scored models from 1,000 independent PyRy3D runs and filtered them based on the ability to fulfil known interactions not included in the initial spatial restraints. In this way, we obtained a cluster of 12 models, from which we selected the representative model with the highest PyRy3D score. This model (Fig. 6a) snugly occupies the available volume of the density map (the cross-correlation coefficient was equal to 0.81).

## Discussion

We carried out a comprehensive structural characterization of the *S. pombe* CCR4-NOT complex, a macromolecular assembly with no symmetry, relatively low stability and probable high flexibility. Despite these limitations, we obtained a medium-resolution (20 Å) structure of the entire complex, which has not yet been obtained using high-resolution techniques such as X-ray crystallography. We located all individual subunits using a variety of methods, from which we built a pseudo-atomic model of the entire assembly in accordance with previous biochemical and structural data; this included the proposed location for nuclease active sites and the binding surfaces of known interacting partners. Our model supports previous functional information and provides new structural data that could be useful for understanding the mechanism of action of the CCR4-NOT complex.

Not1 is a large protein that acts as a scaffold for the complex and spans its entire volume (Fig. 6 and Supplementary Fig. 7). The N-terminal alpha-helical segment (residues 36–815), located in the shorter arm of the 3D reconstruction, forms a platform accessible for protein–protein interactions. The central part of *S. cerevisiae* Not1 interacts with the nuclease module (residues 754–1,000, called the MIF4G domain) and with Caf40 (residues 1,071–1,282, Caf40-binding domain)^8^,^9^. Caf40, located within CCR4-NOT, indicated the upper region of the long arm of the L-shaped complex near the Not2 and Not3 subunits. Azzouz *et al*.^43^ showed that Caf40 and Not5 (the yeast Not3 orthologue) are involved in nuclear RNA quality control by physical and functional connection to the nuclear exosome and the TRAMP complex; the proximity of the subunits reported here further supports these findings. A role was recently reported for human Caf40 in recruitment of GW182 to the miRNA silencing machinery^8^. The proximity of Caf40 to Caf1 deadenylase (∼50 Å from the Caf1 active site) bears out recent experiments showing that Caf40 recruits GW182 protein and the miRNA targets to the CCR4-NOT to initiate its degradation through deadenylation^8^,^9^. The NOT1 Caf1-binding MIF4G domain can interact simultaneously with DDX6, a DEAD-box helicase that further recruits the decapping factors and represses translation^8^,^9^. The known crystal structure of Not1-DDX6 was superimposed on the pseudo-atomic model of the CCR4-NOT complex (Supplementary Fig. 8). DDX6 and Caf1 were positioned on the opposite sites of the Not1 MIF4G domain. This spatial separation provides a suitable platform for binding the mRNA molecules, whose 3′ poly(A) tail is degraded by the Caf1 nuclease, while the 5′ cap-containing end is bound by DDX6 and possibly by other decapping factors (Fig. 6b,c). Deadenylation is thought to occur first, followed by decapping^44^; the structural information reported here suggests that these processes could be simultaneous, in agreement with co-translational decapping.

The molecular architecture of the CCR4-NOT complex suggests that the nuclease module is located near the *S. pombe*-specific subunit Mmi1. Although Mmi1 function in the regulation of *S. pombe* meiosis is well established, the reason it is incorporated into CCR4-NOT and its functional implications are less clear. The fact that Mmi1 is a CCR4-NOT component suggests a role for the complex in the decay of Mmi1-bound meiotic mRNA. The electrostatic potential calculation showed a positively charged patch in the region where Mmi1 is located; in accordance with the general Mmi1 function in the elimination of meiotic-specific mRNA^45^; we pinpointed this as a possible mRNA-binding region (Supplementary Fig. 9). Mmi1 is also involved in facultative heterochromatin formation mediated by the RITS (RNA-induced transcriptional silencing) complex^46^,^47^,^48^ and interestingly Mmi1 association with the CCR4-NOT complex is essential for this process^12^. Although no GW182 homologue has been characterized in *S. pombe*, another GW repeat-containing protein, Tas3, is involved in RITS formation^49^. Caf40 proximity to the Not module thus suggests that, in analogy to *D. melanogaster*, the interaction of GW repeat-containing protein in *S. pombe* includes additional CCR4-NOT complex components. RITS is composed of Ago1 (which binds siRNA), the chromodomain protein Chp1 and Tas3. Mmi1 guides RNAi to specific meiotic mRNAs and genes^47^, although direct physical interaction between RITS components and Mmi1 has not been shown. It is thus possible that CCR4-NOT complex components such as Caf40 or Not2 mediate RITS recruitment to the meiotic genes. Moreover, RITS recognizes methylation on lysine 9 of histone 3 (ref. 47), which provides a link to the second enzymatic activity of the complex, ubiquitination. Not4 (an E3 ubiquitin ligase), which in our structural model is located near Mmi1 and Caf40 (Fig. 6), regulates histone modifications indirectly through degradation of the histone dimethylase Jdh2 (ref. 50). This spatial proximity explains the finding that Not4 is indeed involved in heterochromatin formation^12^. ChIP-seq experiments showed a striking reduction in H3K9 dimethylation in the heterochromatin regions of *not4* mutants^12^. The organization of CCR4-NOT complex subunits illustrates the cooperation between the CCR4-NOT complex, Mmi1 and RITS in *S. pombe* sexual development.

In man, the central part of Not1 (residues 820–999) binds TTP, which in turn binds the 3′-untranslated region AU-rich region on the target mRNA, leading to its deadenylation and decay^13^. Our structural information aids understanding of the underlying mechanisms of this process (Fig. 6c). In this model, the mRNA molecule is bound to TTP and is thus positioned near the active site of the Caf1 nuclease, which mediates deadenylation of the mRNA. It is tempting to speculate that DDX6 helicase, located nearby, binds simultaneously to the 5′ end of the mRNA and represses its translation, induces decapping and further degradation.

The HEAT domain in the scaffolding Not1 C terminus binds cooperatively to the NOT boxes located in the two Not2-Not5 dimer proteins. The entire module is located in the long arm of the L-shaped complex (Figs 5 and 6). The NOT boxes occupy the space at the top of the volume, and the remainder of Not3 stretches downwards at the back of the complex, and with Not1, forms an accessible binding platform. Analysis of CCR4-NOT electrostatic potential (Supplementary Fig. 9) and previous biochemical data^7^,^51^ suggest RNA-binding properties of the Not module, which might be responsible for recruitment of the complex to mRNA targets. Not4, which is involved in protein quality control, interacts with the C terminus of the yeast Not1 (refs 6, 29). The binding region shows an extensive interaction network between Not4 and Not1, which is independent of the Not module^29^. In our pseudo-atomic model, Not4 was positioned at the central part and accessible back side of the volume, between the nuclease and Not modules. The relative proximity of Not4 to the nuclease module of the complex on one hand contradicts the finding that Not4 enzymatic activity is independent of deadenylation^5^. On the other hand, it supports recent results showing that in humans, the E3 ligase MEX-3C regulates Cnot7 deadenylation activity by ubiquitination, but not by degradation^52^. By analogy, yeast Not4 might also have a role in deadenylase activation in the CCR4-NOT complex.

Finally, based on this pseudo-atomic model, the Not1 segment whose structure and function have not yet been characterized (amino acids ∼1,500–1,850), is located at the back of the 3D reconstruction. In this position, an open, accessible surface is formed for potential protein–protein interactions as well as a binding site for the regulatory factors.

In summary, we propose the molecular architecture of the CCR4-NOT complex and outline possible mechanistic aspects that could assist functional studies that will improve understanding of the machinery that controls so vital a process as mRNA processing within the cell.

## Methods

### Yeast strains

The *S. pombe* strains used are listed in Supplementary Table 1. The transformation procedure followed was as described^53^. A pku80Δ strain was used for transformation to minimize non-homologous recombination^53^. To generate spores, strains were crossed on EMM-N plates, resuspended in sterile water, incubated (30 min, 55 °C) and streaked on selective media^54^.

### Purification of the CCR4-NOT complex

CCR4-NOT was purified in two steps. The first used a modified tandem affinity purification procedure^55^,^56^ with Protein A as an affinity tag and the TEV protease cleavage site (ProtA-TEV-Not2) fused to the bait protein (Not2 subunit C terminus). *S. pombe* cells were cultured in 4 l of × 4 YE medium to A_600_=8. Cells were collected by centrifugation (2,400*g*, 4 min, 4 °C), washed with water, the pellet resuspended (100 g 30 ml^−1^) in storage buffer (50 mM HEPES, pH 8.0, 1 mM ditheiothreitol), frozen by plunging into liquid nitrogen and stored at −80 °C. To extract proteins, frozen yeast cells were disrupted mechanically in a laboratory blender cooled with dry ice to avoid sample melting. Cells were melted on ice in lysis buffer (1 mM ditheiothreitol, 40 mM Hepes, pH 8.0, 1 M NaCl, two tablets EDTA-free complete protease inhibitor cocktail, 2 mM phenylmethanesulfonylfluoride (50 ml 100 g^−1^). The homogenate was clarified by centrifugation (48,000*g*, 20 min, 4 °C) and the supernatant centrifuged (131,000*g*, 40 min, 4 °C). The extract was dialyzed (3 h, 4 °C) against buffer D (1 mM ditheiothreitol, 40 mM HEPES, pH 8.0, 0.5 M NaCl, 1 mM phenylmethanesulfonylfluoride, 20% glycerol) using 16 mm dialysis tubes (Merck). Dialyzed protein extract was incubated (overnight, 4 °C) with 1 ml IgG beads (IgG SepharoseTM 6 Fast Flow, GE Healthcare), then centrifuged (2,400*g*, 4 min, 4 °C). Beads were transferred into a poly-prep chromatography column (Bio-Rad), washed with 30 ml IPP500 buffer (500 mM NaCl, 10 mM Tris-HCl, pH 8.0, 0.1% Triton X100), followed by washing with 20 ml TEV protease buffer (500 mM NaCl, 10 mM Tris-HCl, pH 8.0, 1 mM ditheiothreitol, 0.5 mM EDTA). CCR4-NOT complex-bound beads were incubated with 60 μl TEV protease (5 mg ml^−1^) in 1 ml TEV buffer (2.5 h, 18 °C). The complex was eluted with 0.8 ml TEV buffer.

The TEV eluate was loaded onto a 10–30% glycerol gradient (10 mM HEPES, pH 8.0, 500 mM NaCl) and centrifuged (274,000*g*, 18 h, 4 °C); gradient fractions were collected starting from the top (300 μl per fraction), after which 150 μl of each fraction was mixed with the same volume of the neighbouring fraction (total 300 μl), precipitated by trichloroacetic acid, resuspended in 20 μl × 5 Laemmli loading buffer (50 mM Tris-HCl, pH 7.6, 2 mM EDTA, 2% (w/v) SDS, 25% glycerol, 0.01% (w/v) bromophenol blue, 5% (v/v) β-mercaptoethanol, in H_2_0) and analyzed by 10% SDS-PAGE. For cryo-EM analysis, the sample was loaded onto a glycerol gradient as described, except that the 30% solution contained 0.15% glutaraldehyde, according to the GraFix procedure^35^.

### Expression and purification of the Not2-Not5 dimer

*S. cerevisiae* Not2 was cloned into a kanamycin-resistant pET28-derived vector as an N-terminal HIS-SUMO tag fusion protein cleavable with the SUMO-specific protease Ulp1. *S. cerevisiae* Not5 was cloned into a pET28-derived vector with an ampicillin resistance marker as an N-terminal Strep-tag fusion protein. Proteins were coexpressed in *E. coli* BL21-RIL in Super Broth Auto Induction Media (Formedium, Norfolk, UK; 48 h, 18 °C), resuspended in lysis buffer (150 mM NaCl, 50 mM Tris, pH 8.0, 10 mM β-mercaptoethanol, 10 mM imidazole, 300 mM urea, 50 mM arginine, 50 mM glutamine) supplemented with protease inhibitors, and lysed with lysozyme in a EmulsiFlex-C3 homogenizer (Avestin Europe GmbH, Mannheim, Germany). The Not2-Not5 heterodimer was purified on a ÄktaXpress FPLC system (GE Healthcare). The following protocol was used for automated purification: (i) nickel affinity chromatography with on-column SUMO protease cleavage (buffer: 150 mM NaCl, 10 mM Tris, pH 8.0, 10 mM β-mercaptoethanol, 10 mM or 600 mM imidazole), (ii) desalting followed by a second round of nickel affinity chromatography, (iii) ion exchange chromatography (RESOURCE Q column, buffer: 100–1,000 mM NaCl gradient, 10 mM Tris, pH 8.0).

### *In vitro* deadenylation assay

A 40 bp RNA oligonucleotide with a poly(A) tail (5′-cgacgauugcaaaaaaaaaaaaaaaaaaaaaaaaaaaaaa-3′), ^32^P-labelled on the 5′ end, was used as a substrate in the *in vitro* deadenylation reaction. The reaction was performed with 10 pmol RNA substrate and ∼1.5 pmol (∼0.1 μg) purified CCR4-NOT complex in reaction buffer (10 mM Tris-Cl pH 8.0, 10 mM ditheiothreitol, 50 mM KCl, 5 mM MgCl_2_, 5 mM MnCl_2_) and incubated at 30 °C. Samples were collected at 0, 15, 30 and 60 min. To terminate the reaction, 5 μl loading buffer were added (7 M urea, 0.1% bromophenol blue in TBE). Reaction products were analyzed by denaturing electrophoresis in a 19% urea-acrylamide gel (1 h, 300 V), and visualized by phosphoimager (Bio-Rad).

### Electron microscopy

*Sample preparation and data collection*. For negative staining experiments, 5 μl aliquots of samples were applied to 400 mesh grids (Maxtaform Cu/Rh HR26) coated with a thin (∼8 nm) carbon layer and glow-discharged for 20 s. The grids were then stained (2 min) with 2% uranyl acetate and air-dried before transmission EM analysis. Images were taken using a JEM 1200 electron microscope operated at 100 kV (JEOL) in low-dose conditions (10 e/Å) at × 60,000 magnification and recorded on Kodak-electron SO-163 film. Micrographs were digitalized using a Photoscan TD Zeiss-Intergraph scanner (pixel size 2.33 Å/px) or in a Super CoolScan 9000 ED (Nikon; pixel size 2.12 Å/px). Alternatively, images were taken with a JEOL 1010 JEM electron microscope operating at 80 kV with a CCD camera (4k × 4k TemCam-F416, TVIPS). Images were recorded at a sampling rate of 2.97 Å/px.

For cryo-EM sample preparation, we used quantifoil grids (Quantifoil R 1.2/ R1.3 300 mesh grids; ref. Q09684) covered with a thin carbon layer (4 nm) and glow-discharged for 20 s. Aliquots (5 μl) of purified concentrated CCR4-NOT were incubated (2–5 min) with the grid, blotted and plunged into a liquid ethane chamber. All operations were performed on a Leica CPC manual plunger. Cryo-EM samples were analyzed with a Tecnai F20 transmission EM (200 kV). Images were acquired under low-dose conditions (10 e Å^−1^) at × 62,000 magnification and defocus values ranging from −2 to −4 μm. A 4k × 4k *FEI* Eagle CCD camera was used for image recording with a sampling rate of 1.78 Å px^−1^.

*Image processing, particle selection and 2D classification*. In all cases, the CTF (contrast transfer function) was corrected using the CTFFIND3 program^57^, which also calculates potential astigmatism. Micrographs with visible drift and astigmatism were discarded. Single particles were selected manually, extracted from micrographs and normalized using the XMIPP software package^58^. Three types of algorithms implemented in XMIPP were used to classify single images, ML2D (ref. 34), CL2D (ref. 33) and KerDenSom for immune complexes^39^.

*3D reconstruction*. Several initial models were tested in the first step of the 3D reconstruction procedure using EMAN software^59^: artificial noise, blob, a model created by a ‘common lines' algorithm based on previously obtained 2D classes and, for 3D reconstruction of the CCR4-NOT complex based on cryo-EM data and ‘Mmi1-delta' data, a negative staining 3D volume low-pass filtered to 40 Å resolution. Refinement was performed until the 3D reconstructions from these initial models converged to stable, similar 3D volumes (Supplementary Fig. 3). To obtain more structural detail, the 3D reconstruction from EMAN refinement was subjected to Projection Matching using XMIPP. Resolution of the final 3D models was estimated based on the FSC criterion (Fourier shell correlation^60^). The spatial frequency at 0.5 correlation was taken as the resolution of the model (Supplementary Fig. 2). Visualization of the 3D models and docking of the atomic structures into EM volumes was performed manually using USCF Chimera^61^.

*Immunoelectron microscopy*. The subunits of the CCR4-NOT complex were located using immunolabeling. Complexes were purified as described above using the Protein A tag in the Not2 subunit. The C terminus of each target subunit (Caf1, Ccr4, Not1, Caf40, Not3/5 or Not4) was fused to GFP. After affinity chromatography using IgG beads and TEV protease cleavage, the TEV eluate was incubated (30 min, 4 °C) with 50 μg anti-GFP monoclonal antibody (1 mg ml^−1^; G6795, Sigma) or a Fab fragment from the same antibody (Mouse IgG1 Fab and F(ab')2 Micro Preparation Kit; Pierce) and loaded onto a glycerol gradient. After separation of gradient fractions and SDS-PAGE analysis, the fraction with the highest concentration and purity of the immune complex (CCR4-NOT+IgG) was analyzed by negative staining EM.

*Nanogold labelling*. A 34-nt RNA oligonucleotide was covalently coupled to Nanogold (ref. 2021; Nanoprobes) containing a primary amine functional group on its surface, reactive to aldehyde groups. In the first step, 500 pmol RNA oligonucleotide were oxidized with 1,000 nM NaIO_4_ in 100 mM PIPES buffer pH 7.0 (90 min, 4 °C). The reaction was terminated by incubation with 2 μl glycerol (5 min). Oxidized RNA was precipitated with 96% cold ethanol. The RNA pellet was resuspended in 250 μl 0.1% glycol ethylene solution to remove excess NaIO_4_ and incubated (30 min, 0 °C). Finally, the RNA product was again precipitated with 96% ethanol.

Aliquots (30 nM) of monoamino Nanogold resuspended in 100 μl dimethyl sulphoxide were incubated with 60 μl oxidized RNA from the previous step in 100 mM PIPES pH 7.0 (60 min, 4 °C, with gentle mixing). Aliquots (3 μl) of freshly prepared sodium borohydride (20 mg ml^−1^) were added to the gold-coupling reaction and incubated (30 min, 4 °C). The reaction was terminated by adding 2 μl acetone. Nanogold-labelled RNA was purified on a S200 Ilustra MicroSpin column (GE Healthcare). Aliquots (1–10 μl) of purified Nanogold-labelled RNA were mixed with 5–10 μl purified CCR4-NOT complex and incubated (30 min, ice). Samples were prepared and analyzed by negative staining EM.

### Pseudo-atomic model building

*Homology modelling*. Homology structures of all CCR4-NOT components were modelled based on the *S. pombe* sequences from the fission yeast database (http://www.pombase.org/). For template selection and domain identification, we used the GeneSilico Metaserver (https://genesilico.pl/meta2/) (ref. 62). Intrinsically disordered regions were identified with MetaDisorder^63^. Comparative models of the CCR4-NOT subunits were built using the ‘FRankenstein's Monster' modelling approach^64^, which comprises cycles of model building, evaluation, realignment of poorly scored regions and merging of the best-scoring fragments to obtain the best possible model. For model building we used MODELLER 8v1 (ref. 65). For regions without a template or poorly modelled, we used a REFINER^66^ program that performs local refinement with restraints on predicted secondary structure. Finally, we built models of Not1-Not2-Not3 (Not1: 1,583–2,072, Not2: 128–306, Not3: 491–634), Not1-Caf1-Ccr4 (Not1: 841–1,090 and Caf1: 23–273 and Ccr4: entire), Not1-Caf40 (Rcd1) (Not1: 1,097–1,328 and Caf40: entire) and Not1-Not4 (Not1: 1,568–2,078, Not4: 420–469) complexes in which specific components were oriented according to 4by6 (ref. 7), 4b8c (ref. 25), 4cru (ref. 8) and 5aje (ref. 29) crystal structures, respectively. All models obtained were evaluated with the MetaMQAPII program^67^. Values of predicted GDT_TS (global distance test total score) were used to evaluate global accuracy of models (the higher the GDT_TS, the better) and the predicted deviation of individual residues as a measure of predicted local accuracy (the lower the predicted deviation, the better). MQAP scores only predict the deviation of a model from the real structure; the real deviation can be calculated only by comparison to the real structures, which are not available for this complex. The scores reported here as ‘very good models' must thus be interpreted as estimations or predictions that our models are ‘very good', of an ultimate validation of model quality. The final models of individual proteins, with quality evaluations, are publicly available at: ftp://ftp.genesilico.pl/iamb/models/ccr4not.

*Complex assembly*. To predict the structure of the CCR4-NOT complex and the orientation of its components within the electron density map, rigid body docking methods were first applied to confirm the positions of the components identified by immunolocalization experiments. The PyRy3D method developed in the Bujnicki laboratory (http://genesilico.pl/pyry3d/, standalone version available from the authors on request) was used to optimize positions obtained based on other data available for the complex.

Rigid body docking was applied to all CCR4-NOT components, according to available crystal structures and models generated in this study. Situs^41^ and ADP_EM^42^ programs were then used, and best-scored solutions that coincided with immunolocalization results for specific components were selected. Both programs were used with default parameters. Map resolution was set to 20 Å and a density threshold to 0.7.

Positions for Ccr4-Caf1-Not1, Caf40-Not1 and Not2-Not3-Not1 modules returned by Situs were used as starting points for conformational space sampling with PyRy3D. Protein sequence regions predicted to be disordered were modelled in a coarse-grained representation and treated as flexible shapes able to change conformation during complex assembly. To define interactions between components of the complex, we used the following constraints:

- Residues 16–76 and 113–200 of Not4 were constrained to be in proximity to the Not1 C terminus (residues 1,326–2,072).
- Mmi1 was constrained to interact with Caf1 (at least one contact between any residues of these proteins was required).
- Mmi1 (any residue) was constrained to be in contact with residues 1–1,200 of Not1.
- The Not1 domains were constrained to remain connected to one another.

PyRy3D was used with default parameters (Monte Carlo simulated annealing method, starting temperature T_0_=10 in dimensionless units, temperature decrease during the simulation according to the scheme: T_n_=T_0_ × 0.999^n^; where n is the number of the simulation steps, 100,000 steps, PyRy3D grid size 1.5 Å). The 0.7 density threshold was used to define the map volume.

A total of 1,000 models of the CCR4-NOT complex were generated from 1,000 runs of PyRy3D; they were clustered according to RMSD values between the models to obtain groups of most similar solutions. The resulting clusters of models were filtered according to their ability to fulfil interactions identified for human proteins (Cnot1-TTP and Nanos1 (pdb:4j8s), Cnot9-W, DDX6 (pdb:4ct7,4ct4), Caf1 with Tob/Btg family proteins (pdb:2d5r) and yeast Not4 with Ubc4 (pdb:4ajd). A group of CCR4-NOT models was selected, in which regions responsible for these interactions were exposed to the solvent. As the final model, we chose the best-scored model (according to PyRy3D score) from the filtered cluster of solutions. Goodness of fit of the models into the electron density map was measured with the cross-correlation coefficient implemented in the ‘Fit In Map' procedure available via the Chimera viewer. The final ensembles of models are available at ftp://ftp.genesilico.pl/iamb/models/ccr4not. Molecular structure graphics were produced with PyMOL^68^ and Chimera^61^.

## Additional information

**Accession codes:** The 3D reconstruction of the CCR4-NOT complex generated by cryo-EM has been deposited in the EMDB (code EMD3232).

**How to cite this article:** Ukleja, M. *et al*. The architecture of the *Schizosaccharomyces pombe* CCR4-NOT complex. *Nat. Commun.* 7:10433 doi: 10.1038/ncomms10433 (2016).

## Supplementary Material

Supplementary Information Supplementary Figures 1-9, Supplementary Tables 1-3.

## References

[b1] Miller J. E. & Reese J. C. Ccr4-Not complex: the control freak of eukaryotic cells. Crit. Rev. Biochem. Mol. Biol. 47, 315–333 (2012). 22416820 10.3109/10409238.2012.667214 PMC3376659

[b2] Collart M. A. & Panasenko O. O. The Ccr4–not complex. Gene 492, 42–53 (2012). 22027279 10.1016/j.gene.2011.09.033

[b3] Collart M. A. & Struhl K. NOT1(CDC39), NOT2(CDC36), NOT3, and NOT4 encode a global-negative regulator of transcription that differentially affects TATA-element utilization. Genes Dev. 8, 525–537 (1994). 7926748 10.1101/gad.8.5.525

[b4] Collart M. A. The Not4 RING E3 ligase: a relevant player in cotranslational quality control. ISRN Mol. Biol. 2013, 548359 (2013). 10.1155/2013/548359 PMC4890865 27335678

[b5] Halter D., Collart M. A. & Panasenko O. O. The Not4 E3 ligase and CCR4 deadenylase play distinct roles in protein quality control. PLoS ONE 9, e86218 (2014). 24465968 10.1371/journal.pone.0086218 PMC3895043

[b6] Bai Y. . The CCR4 and CAF1 proteins of the CCR4-NOT complex are physically and functionally separated from NOT2, NOT4, and NOT5. Mol. Cell Biol. 19, 6642–6651 (1999). 10490603 10.1128/mcb.19.10.6642 PMC84645

[b7] Bhaskar V. . Structure and RNA-binding properties of the Not1-Not2-Not5 module of the yeast Ccr4-Not complex. Nat. Struct. Mol. Biol. 20, 1281–1288 (2013). 24121231 10.1038/nsmb.2686

[b8] Chen Y. . A DDX6-CNOT1 complex and W-binding pockets in CNOT9 reveal direct links between miRNA target recognition and silencing. Mol. Cell 54, 737–750 (2014). 24768540 10.1016/j.molcel.2014.03.034

[b9] Mathys H. . Structural and biochemical insights to the role of the CCR4-NOT complex and DDX6 ATPase in microRNA repression. Mol. Cell 54, 751–765 (2014). 24768538 10.1016/j.molcel.2014.03.036

[b10] Bawankar P., Loh B., Wohlbold L., Schmidt S. & Izaurralde E. NOT10 and C2orf29/NOT11 form a conserved module of the CCR4-NOT complex that docks onto the NOT1 N-terminal domain. RNA Biol. 10, 228–244 (2013). 23303381 10.4161/rna.23018 PMC3594282

[b11] Mauxion F., Prève B. & Séraphin B. C2ORF29/CNOT11 and CNOT10 form a new module of the CCR4-NOT complex. RNA Biol. 10, 267–276 (2013). 23232451 10.4161/rna.23065 PMC3594285

[b12] Cotobal C. . Role of Ccr4-Not complex in heterochromatin formation at meiotic genes and subtelomeres in fission yeast. Epigenetics Chromatin 8, 28 (2015). 26279681 10.1186/s13072-015-0018-4 PMC4536793

[b13] Fabian M. R. . Structural basis for the recruitment of the human CCR4–NOT deadenylase complex by tristetraprolin. Nat. Struct. Mol. Biol. 20, 735–739 (2013). 23644599 10.1038/nsmb.2572 PMC4811204

[b14] Bhandari D., Raisch T., Weichenrieder O., Jonas S. & Izaurralde E. Structural basis for the Nanos-mediated recruitment of the CCR4-NOT complex and translational repression. Genes Dev. 28, 888–901 (2014). 24736845 10.1101/gad.237289.113 PMC4003280

[b15] Doidge R., Mittal S., Aslam A. & Winkler G. S. Deadenylation of cytoplasmic mRNA by the mammalian Ccr4-Not complex. Biochem. Soc. Trans. 40, 896–901 (2012). 22817755 10.1042/BST20120074

[b16] Villanyi Z. . The Not5 subunit of the Ccr4-Not complex connects transcription and translation. PLoS Genet. 10, e1004569 (2014). 25340856 10.1371/journal.pgen.1004569 PMC4207488

[b17] Babbarwal V., Fu J. & Reese J. C. The Rpb4/7 module of RNA polymerase II is required for carbon catabolite repressor protein 4-negative on TATA (Ccr4-Not) complex to promote elongation. J. Biol. Chem. 289, 33125–33130 (2014). 25315781 10.1074/jbc.C114.601088 PMC4246073

[b18] Deluen C. . The Ccr4-Not complex and yTAF1 (yTafII130p/yTafII145p) show physical and functional interactions. Mol. Cell Biol. 22, 6735–6749 (2002). 12215531 10.1128/MCB.22.19.6735-6749.2002 PMC134042

[b19] Kruk J. A., Dutta A., Fu J., Gilmour D. S. & Reese J. C. The multifunctional Ccr4-Not complex directly promotes transcription elongation. Genes Dev. 25, 581–593 (2011). 21406554 10.1101/gad.2020911 PMC3059832

[b20] Albert T. K. . Identification of a ubiquitin-protein ligase subunit within the CCR4-NOT transcription repressor complex. EMBO J. 21, 355–364 (2002). 11823428 10.1093/emboj/21.3.355 PMC125831

[b21] Panasenko O. O. & Collart M. A. Not4 E3 ligase contributes to proteasome assembly and functional integrity in part through Ecm29. Mol. Cell Biol. 31, 1610–1623 (2011). 21321079 10.1128/MCB.01210-10 PMC3126335

[b22] Xu K., Bai Y., Zhang A., Zhang Q. & Bartlam M. G. Insights into the structure and architecture of the CCR4–NOT complex. Front. Genet. 5, 137 (2014). 24904637 10.3389/fgene.2014.00137 PMC4032980

[b23] Wang H. . Crystal structure of the human CNOT6L nuclease domain reveals strict poly(A) substrate specificity. EMBO J. 29, 2566–2576 (2010). 20628353 10.1038/emboj.2010.152 PMC2928688

[b24] Hanzawa H. . The Structure of the C4C4RING finger of human NOT4 reveals features distinct from those of C3HC4 RING fingers. J. Biol. Chem. 276, 10185–10190 (2001). 11087754 10.1074/jbc.M009298200

[b25] Basquin J. . Architecture of the nuclease module of the yeast Ccr4-Not complex: the Not1-Caf1-Ccr4 interaction. Mol. Cell 48, 207–218 (2012). 22959269 10.1016/j.molcel.2012.08.014

[b26] Andersen K. R., Jonstrup A. T., Van L. B. & Brodersen D. E. The activity and selectivity of fission yeast Pop2p are affected by a high affinity for Zn2+ and Mn2+ in the active site. RNA. 15, 850–861 (2009). 19307292 10.1261/rna.1489409 PMC2673079

[b27] Jonstrup A. T., Andersen K. R., Van L. B. & Brodersen D. E. The 1.4-? crystal structure of the S. pombe Pop2p deadenylase subunit unveils the configuration of an active enzyme. Nucleic Acids Res. 35, 3153–3164 (2007). 17452359 10.1093/nar/gkm178 PMC1888821

[b28] Garces R. G., Gillon W. & Pai E. F. Atomic model of human Rcd-1 reveals an armadillo-like-repeat protein with *in vitro* nucleic acid binding properties. Protein Sci. 16, 176–188 (2007). 17189474 10.1110/ps.062600507 PMC2203284

[b29] Bhaskar V., Basquin J. & Conti E. Architecture of the ubiquitylation module of the yeast Ccr4-Not complex. Structure. 23, 921–928 (2015). 25914052 10.1016/j.str.2015.03.011 PMC4431670

[b30] Nasertorabi F., Batisse C., Diepholz M., Suck D. & Böttcher B. Insights into the structure of the CCR4-NOT complex by electron microscopy. FEBS Lett. 585, 2182–2186 (2011). 21669201 10.1016/j.febslet.2011.05.071 PMC3171648

[b31] Pena A. . Architecture and nucleic acids recognition mechanism of the THO complex, an mRNP assembly factor. EMBO J. 31, 1605–1616 (2012). 22314234 10.1038/emboj.2012.10 PMC3321177

[b32] Alvira S. . Structural characterization of the substrate transfer mechanism in Hsp70/Hsp90 folding machinery mediated by Hop. Nat. Commun. 5, 5484 (2014). 25407331 10.1038/ncomms6484

[b33] Sorzano C. O. S. . A clustering approach to multireference alignment of single-particle projections in electron microscopy. J. Struct. Biol. 171, 197–206 (2010). 20362059 10.1016/j.jsb.2010.03.011 PMC2893300

[b34] Scheres S. H. W. . Maximum-likelihood multi-reference refinement for electron microscopy images. J. Mol. Biol. 348, 139–149 (2005). 15808859 10.1016/j.jmb.2005.02.031

[b35] Stark H. GraFix: stabilization of fragile macromolecular complexes for single particle cryo-EM. Meth. Enzymol. 481, 109–126 (2010). 20887855 10.1016/S0076-6879(10)81005-5

[b36] Temme C. . Subunits of the *Drosophila* CCR4-NOT complex and their roles in mRNA deadenylation. RNA. 16, 1356–1370 (2010). 20504953 10.1261/rna.2145110 PMC2885685

[b37] Chen J. . Purification and characterization of the 1.0 MDa CCR4-NOT complex identifies two novel components of the complex. J. Mol. Biol. 314, 683–694 (2001). 11733989 10.1006/jmbi.2001.5162

[b38] Yamashita A. . Hexanucleotide motifs mediate recruitment of the RNA elimination machinery to silent meiotic genes. Open Biol. 2, 120014 (2012). 22645662 10.1098/rsob.120014 PMC3352096

[b39] Pascual-Montano A. . A novel neural network technique for analysis and classification of EM single-particle images. J. Struct. Biol. 133, 233–245 (2001). 11472094 10.1006/jsbi.2001.4369

[b40] Collart M. A., Panasenko O. O. & Nikolaev S. I. The Not3/5 subunit of the Ccr4-Not complex: a central regulator of gene expression that integrates signals between the cytoplasm and the nucleus in eukaryotic cells. Cell Signal 25, 743–751 (2013). 23280189 10.1016/j.cellsig.2012.12.018

[b41] Wriggers W. Using Situs for the integration of multi-resolution structures. Biophys. Rev. 2, 21–27 (2010). 20174447 10.1007/s12551-009-0026-3 PMC2821521

[b42] Garzón J. I., Kovacs J., Abagyan R. & Chacón P. ADP_EM: fast exhaustive multi-resolution docking for high-throughput coverage. Bioinformatics. 23, 427–433 (2007). 17150992 10.1093/bioinformatics/btl625

[b43] Azzouz N., Panasenko O. O., Colau G. & Collart M. A. The CCR4-NOT complex physically and functionally interacts with TRAMP and the nuclear exosome. PLoS ONE 4, e6760 (2009). 19707589 10.1371/journal.pone.0006760 PMC2727002

[b44] Jonas S. & Izaurralde E. The role of disordered protein regions in the assembly of decapping complexes and RNP granules. Genes Dev. 27, 2628–2641 (2013). 24352420 10.1101/gad.227843.113 PMC3877753

[b45] Chen H.-M., Futcher B. & Leatherwood J. The fission yeast RNA binding protein Mmi1 regulates meiotic genes by controlling intron specific splicing and polyadenylation coupled RNA turnover. PLoS ONE 6, e26804 (2011). 22046364 10.1371/journal.pone.0026804 PMC3203177

[b46] Tashiro S., Asano T., Kanoh J. & Ishikawa F. Transcription-induced chromatin association of RNA surveillance factors mediates facultative heterochromatin formation in fission yeast. Genes Cells 18, 327–339 (2013). 23388053 10.1111/gtc.12038

[b47] Hiriart E. . Mmi1 RNA surveillance machinery directs RNAi complex RITS to specific meiotic genes in fission yeast. EMBO J. 31, 2296–2308 (2012). 22522705 10.1038/emboj.2012.105 PMC3364741

[b48] Holm L. R. & Thon G. New romance between RNA degradation pathways: Mmi1 and RNAi meet on heterochromatic islands. EMBO J. 31, 2242–2243 (2012). 22549465 10.1038/emboj.2012.138 PMC3364736

[b49] Eulalio A., Tritschler F. & Izaurralde E. The GW182 protein family in animal cells: New insights into domains required for miRNA-mediated gene silencing. RNA 15, 1433–1442 (2009). 19535464 10.1261/rna.1703809 PMC2714752

[b50] Mersman D. P., Du H.-N., Fingerman I. M., South P. F. & Briggs S. D. Polyubiquitination of the demethylase Jhd2 controls histone methylation and gene expression. Genes Dev. 23, 951–962 (2009). 19346402 10.1101/gad.1769209 PMC2675863

[b51] Boland A. . Structure and assembly of the NOT module of the human CCR4–NOT complex. Nat. Struct. Mol. Biol. 20, 1289–1297 (2013). 24121232 10.1038/nsmb.2681

[b52] Cano F., Rapiteanu R., Sebastiaan Winkler G. & Lehner P. J. A non-proteolytic role for ubiquitin in deadenylation of MHC-I mRNA by the RNA-binding E3-ligase MEX-3C. Nat. Commun. 6, 8670 (2015). 26471122 10.1038/ncomms9670 PMC4617606

[b53] Fennessy D. . Extending the *Schizosaccharomyces pombe* molecular genetic toolbox. PLoS ONE 9, e97683 (2014). 24848109 10.1371/journal.pone.0097683 PMC4029729

[b54] Khare A. K., Singh B. & Singh J. A fast and inexpensive method for random spore analysis in *Schizosaccharomyces pombe*. Yeast. 28, 527–533 (2011). 21547948 10.1002/yea.1855

[b55] Rigaut G. . A generic protein purification method for protein complex characterization and proteome exploration. Nat. Biotechnol. 17, 1030–1032 (1999). 10504710 10.1038/13732

[b56] Puig O. . The tandem affinity purification (TAP) method: a general procedure of protein complex purification. Methods. 24, 218–229 (2001). 11403571 10.1006/meth.2001.1183

[b57] Mindell J. A. & Grigorieff N. Accurate determination of local defocus and specimen tilt in electron microscopy. J. Struct. Biol. 142, 334–347 (2003). 12781660 10.1016/s1047-8477(03)00069-8

[b58] Marabini R. . Xmipp: an image processing package for electron microscopy. J. Struct. Biol. 116, 237–240 (1996). 8812978 10.1006/jsbi.1996.0036

[b59] Ludtke S. J., Baldwin P. R. & Chiu W. EMAN: semiautomated software for high-resolution single-particle reconstructions. J. Struct. Biol. 128, 82–97 (1999). 10600563 10.1006/jsbi.1999.4174

[b60] van Heel M. & Schatz M. Fourier shell correlation threshold criteria. J. Struct. Biol. 151, 250–262 (2005). 16125414 10.1016/j.jsb.2005.05.009

[b61] Pettersen E. F. . UCSF Chimera–a visualization system for exploratory research and analysis. J. Comput. Chem. 25, 1605–1612 (2004). 15264254 10.1002/jcc.20084

[b62] Kurowski M. A. & Bujnicki J. M. GeneSilico protein structure prediction meta-server. Nucleic. Acids. Res. 31, 3305–3307 (2003). 12824313 10.1093/nar/gkg557 PMC168964

[b63] Kozlowski L. P. & Bujnicki J. M. MetaDisorder: a meta-server for the prediction of intrinsic disorder in proteins. BMC Bioinformatics 13, 111 (2012). 22624656 10.1186/1471-2105-13-111 PMC3465245

[b64] Kosinski J. . A ‘FRankenstein's monster' approach to comparative modeling: merging the finest fragments of Fold-Recognition models and iterative model refinement aided by 3D structure evaluation. Proteins 53, (Suppl 6): 369–379 (2003). 14579325 10.1002/prot.10545

[b65] Webb B. & Sali A. Protein structure modeling with MODELLER. Methods Mol. Biol. 1137, 1–15 (2014). 24573470 10.1007/978-1-4939-0366-5_1

[b66] Boniecki M., Rotkiewicz P., Skolnick J. & Kolinski A. Protein fragment reconstruction using various modeling techniques. J. Comput. Aided Mol. Des. 17, 725–738 (2003). 15072433 10.1023/b:jcam.0000017486.83645.a0

[b67] Pawlowski M., Gajda M. J., Matlak R. & Bujnicki J. M. MetaMQAP: a meta-server for the quality assessment of protein models. BMC Bioinformatics 9, 403 (2008). 18823532 10.1186/1471-2105-9-403 PMC2573893

[b68] Lua R. C. & Lichtarge O. PyETV: a PyMOL evolutionary trace viewer to analyze functional site predictions in protein complexes. Bioinformatics. 26, 2981–2982 (2010). 20929911 10.1093/bioinformatics/btq566 PMC2982157

