# Supplementary material for: The architecture of the Schizosaccharomyces pombe CCR4-NOT complex
Source: Nat Commun. 2016 Jan 25;7:10433. doi: 10.1038/ncomms10433 (PMC4737751; doi:10.1038/ncomms10433)
Supplement: Supplementary Information — Supplementary Figures 1-9, Supplementary Tables 1-3. [file ncomms10433-s1.pdf]

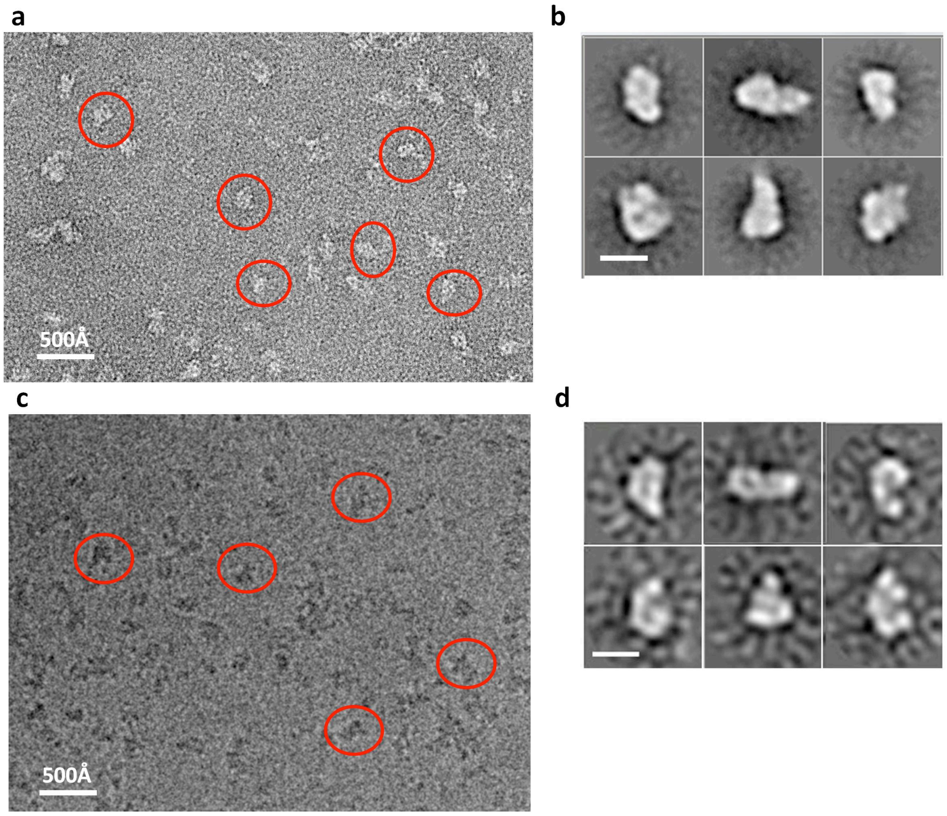

Supplementary Figure 1. Negative staining and cryo-EM analysis of the CCR4-NOT complex. a, c) Example of negative staining (a) and cryo-EM (c) images of the CCR4-NOT complex with some selected individual particles (red circles). **b, d)** Example of the representative reference-free 2D classes of the two preparations. Bar in b,d) = 100Å.

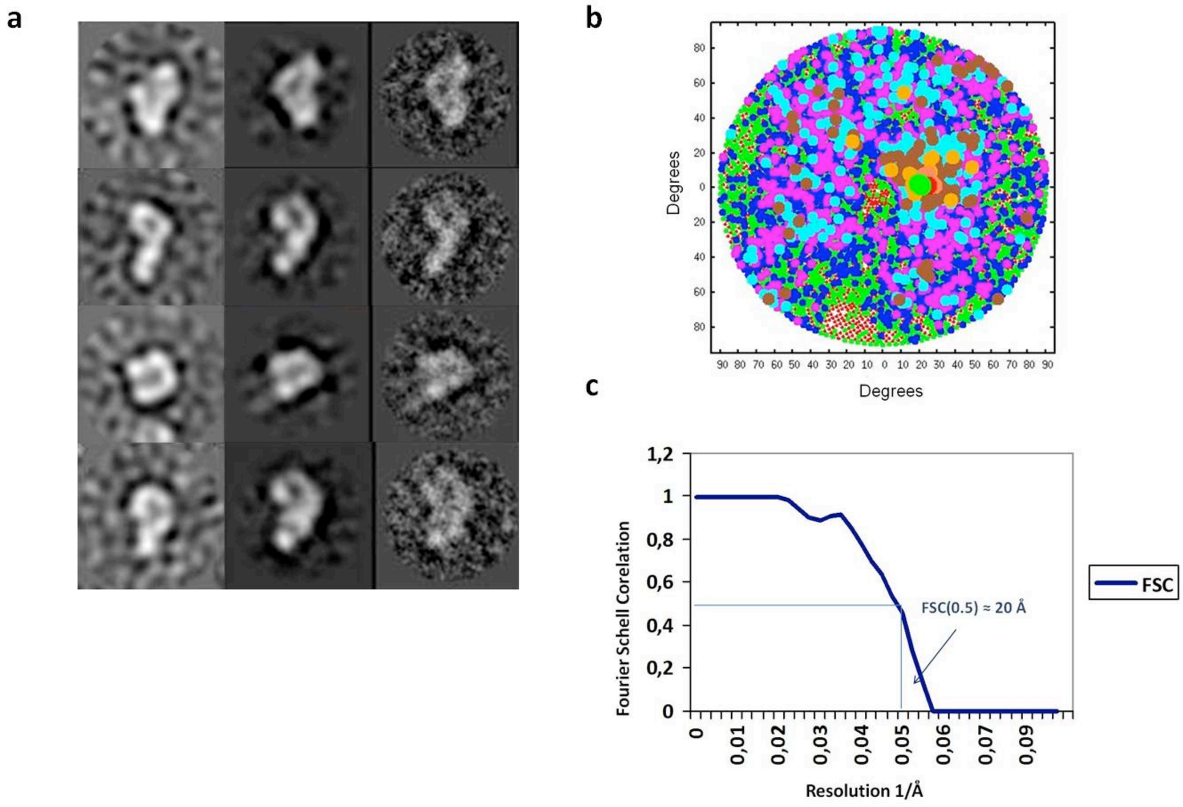

Supplementary Figure 2. Additional assessment of the quality of the 3D reconstructions. **a)** The 3D reconstruction quality can be assessed by comparison of the reference-free 2D classes (CL2D, left column), with the 3D map projections and the generated class averages from EMAN (middle and right, respectively), based on the 3D reconstruction of the CCR4-NOT complex. **b)** 2D representation of the angular distribution of the particles used for the 3D reconstruction of the CCR4-NOT complex by Projection Matching. Each circle represents one projection of the final model with the defined angle value. The size is proportional to the number of assigned individual particles. **c)** The computed resolution curve for the CCR4-NOT complex reconstruction. The resolution was estimated to be 20 Å based on the 0.5 criterion.

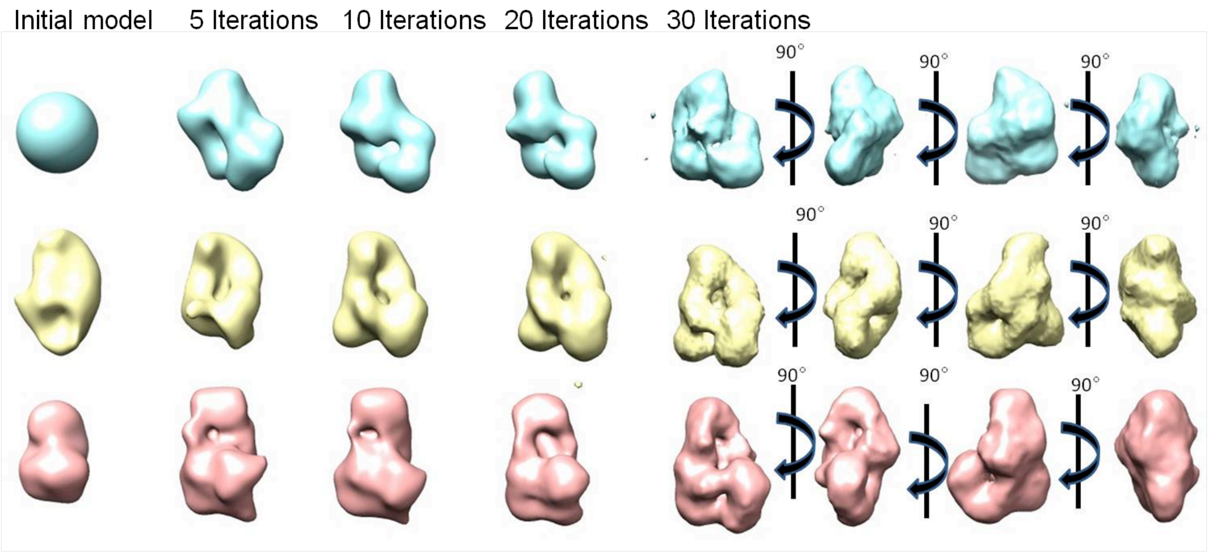

Supplementary Figure 3. Representation of the iterative angular refinement from the 3D reconstruction of CCR4-NOT using the cryoEM images. The iterative refinement was performed simultaneously using EMAN, starting from different initial models: blob (blue), "common lines" (yellow) and a highly-filtered volume of the negative staining 3D reconstructions (pink).

Ccr4-not wt

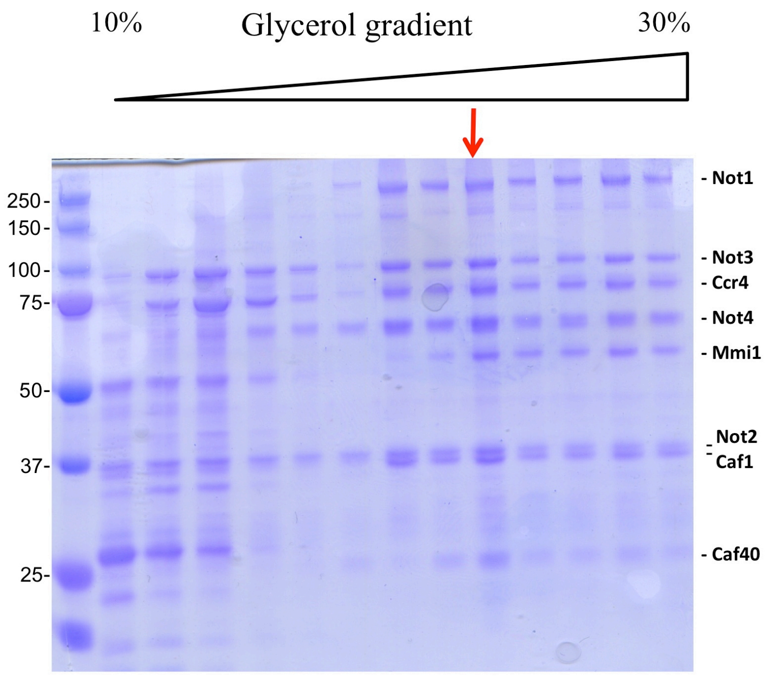

ΔMmi1

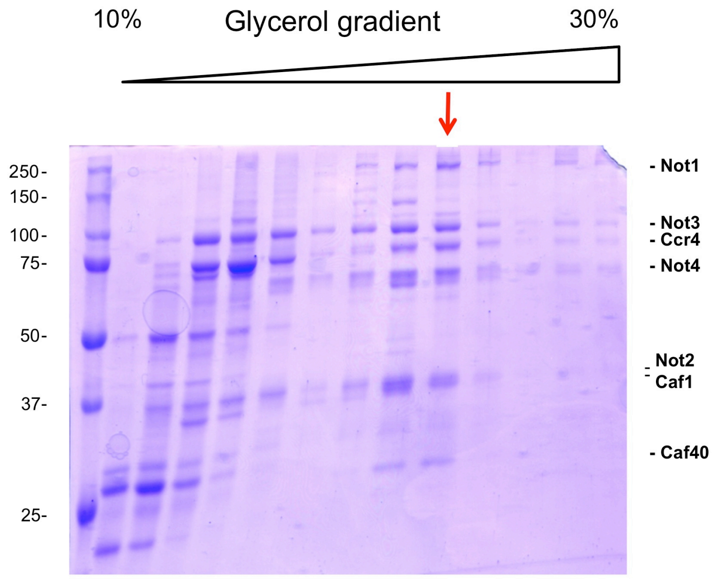

Not3-GFP

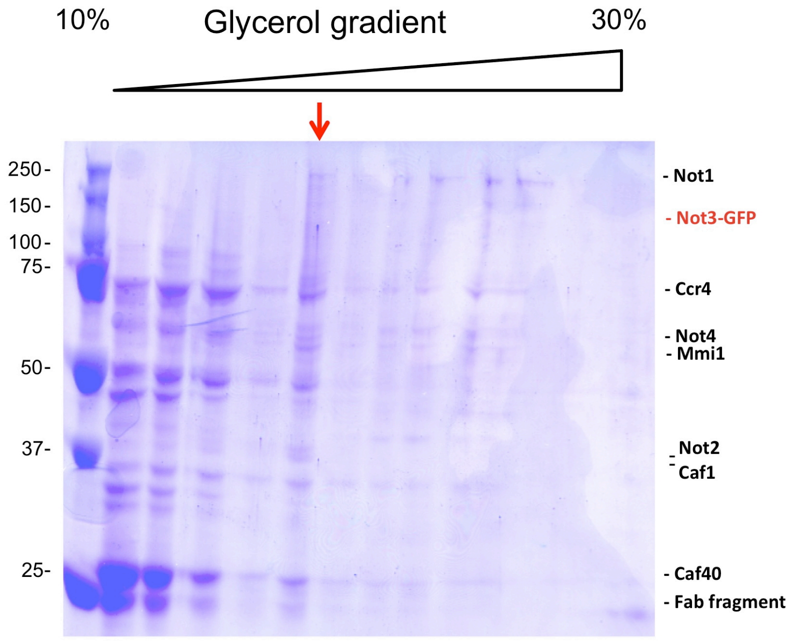

Not1-GFP

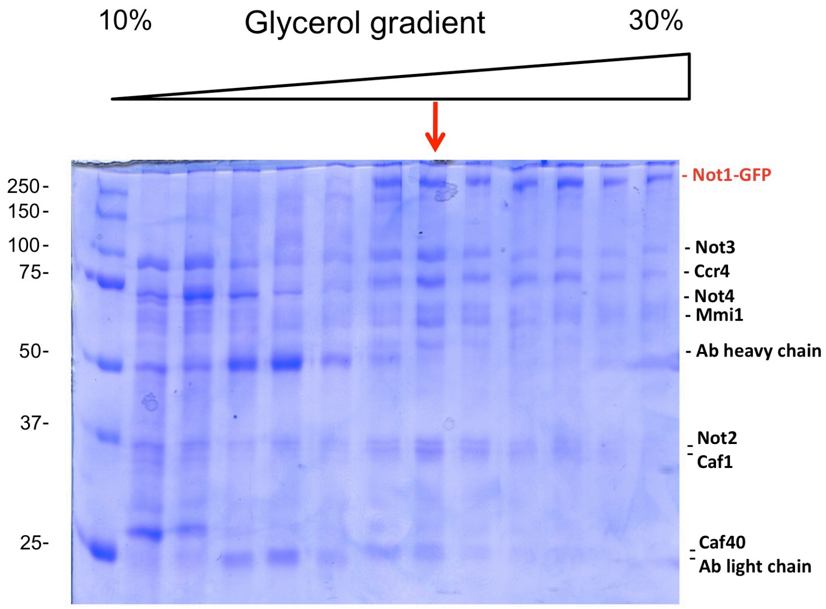

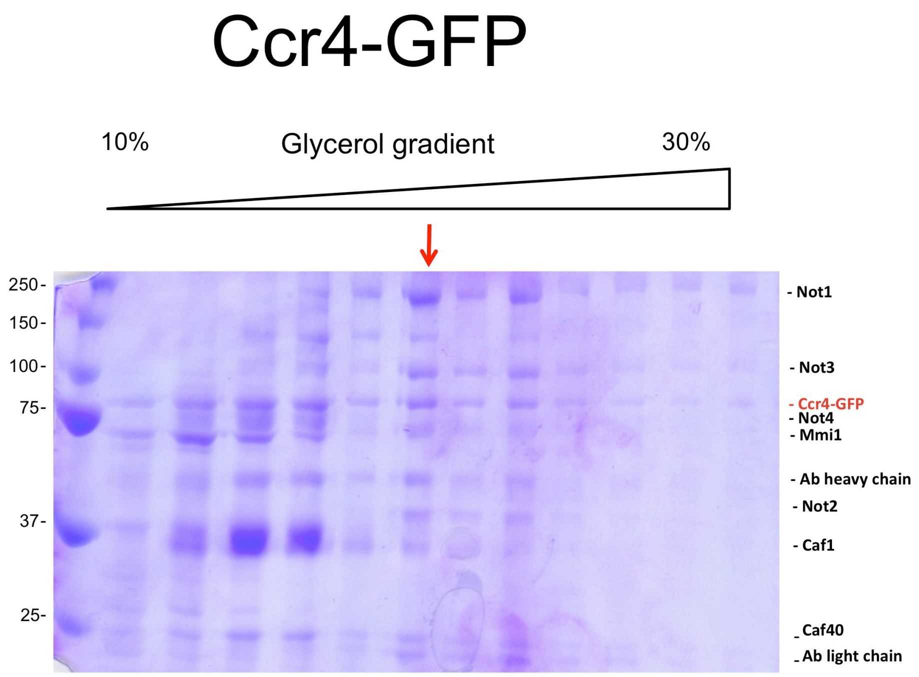
Ccr4-GFP
10%
Glycerol gradient
30%
250-
150-
100-
75-
37-
25-
- Not1
- Not3
- Ccr4-GFP
- Not4
- Mmi1
- Ab heavy chain
- Not2
- Caf1
- Caf40
- Ab light chain

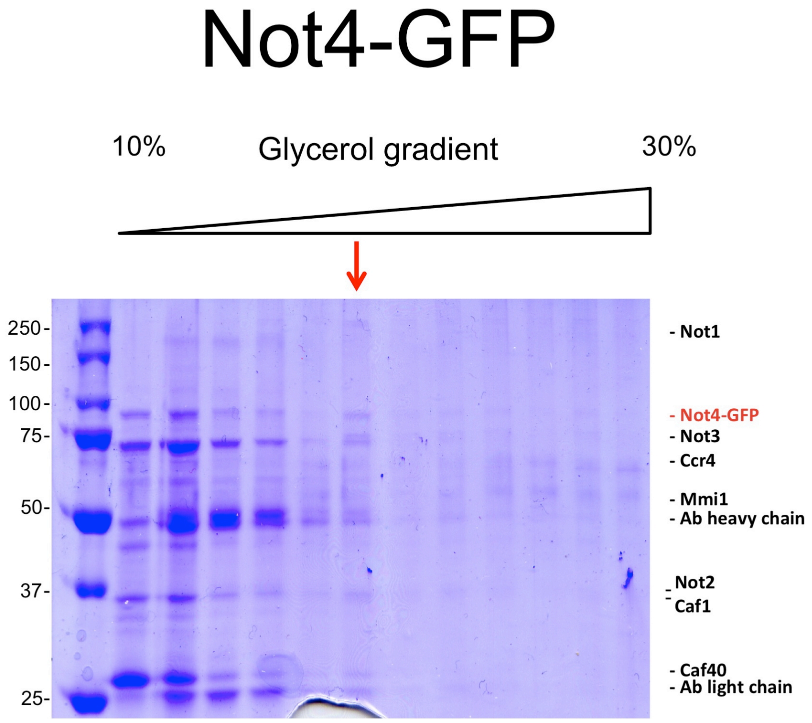
Not4-GFP
10%
Glycerol gradient
30%
250-
150-
100-
75-
50-
37-
25-
- Not1
- Not4-GFP
- Not3
- Ccr4
- Mmi1
- Ab heavy chain
Not2
Caf1
- Caf40
- Ab light chain

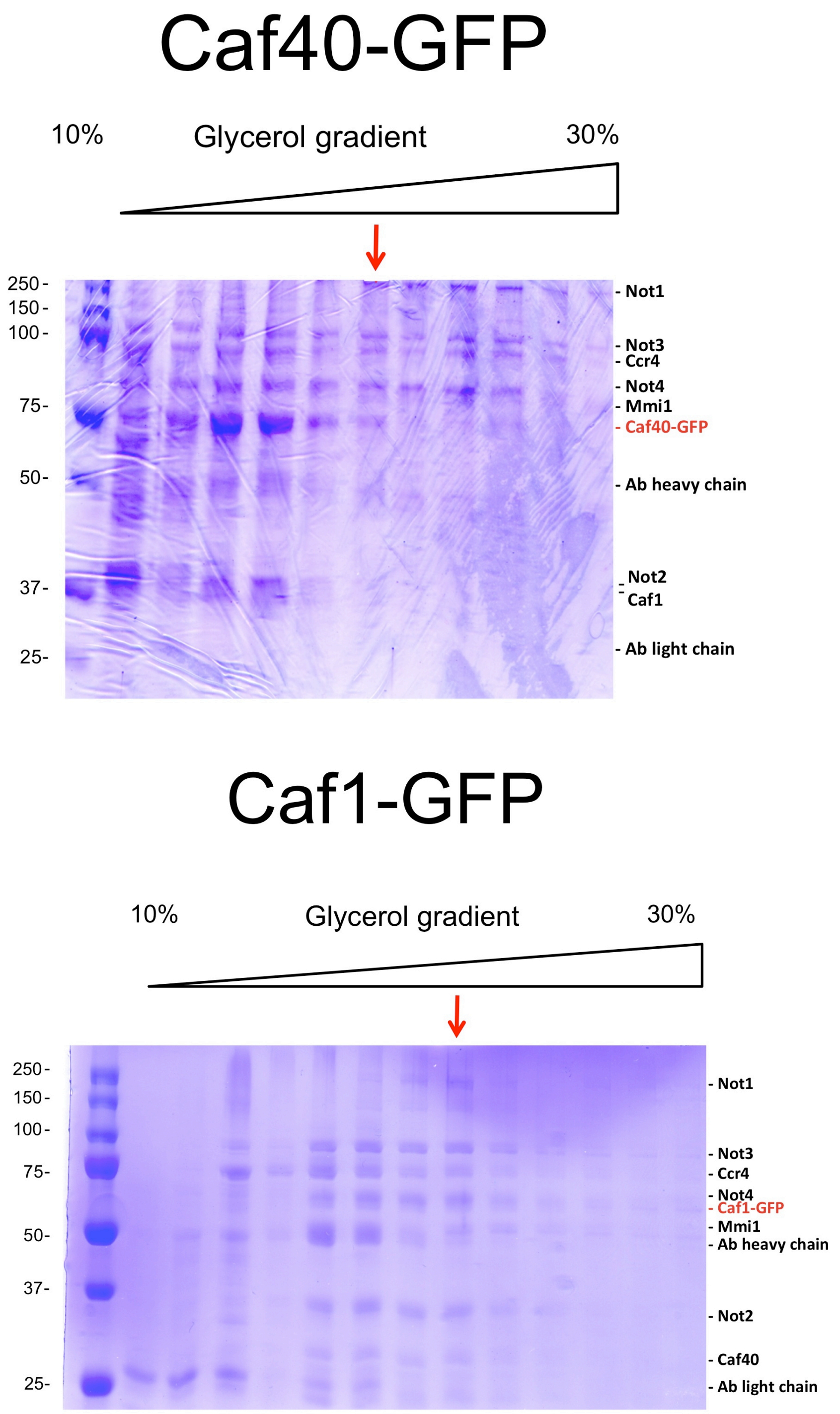

Supplementary Figure 4. Purification of CCR4-NOT complexes from *Schizosaccharomyces pombe.* Electrophoretic analysis of the fractions of the glycerol gradients shown in Fig. 1a (the two first gels) and those used for immunolocalization EM. For immunomicroscopy, the CCR4-NOT complex was purified from distinct yeast strains in which different CCR4-NOT subunits were individually fused to a GFP tag and the Not2 subunit in each strain was fused to a TAP tag. The presence of all CCR4-NOT complex components and antibody heavy and light chains are indicated. The lane marked with a red arrow was used for electron microscopy

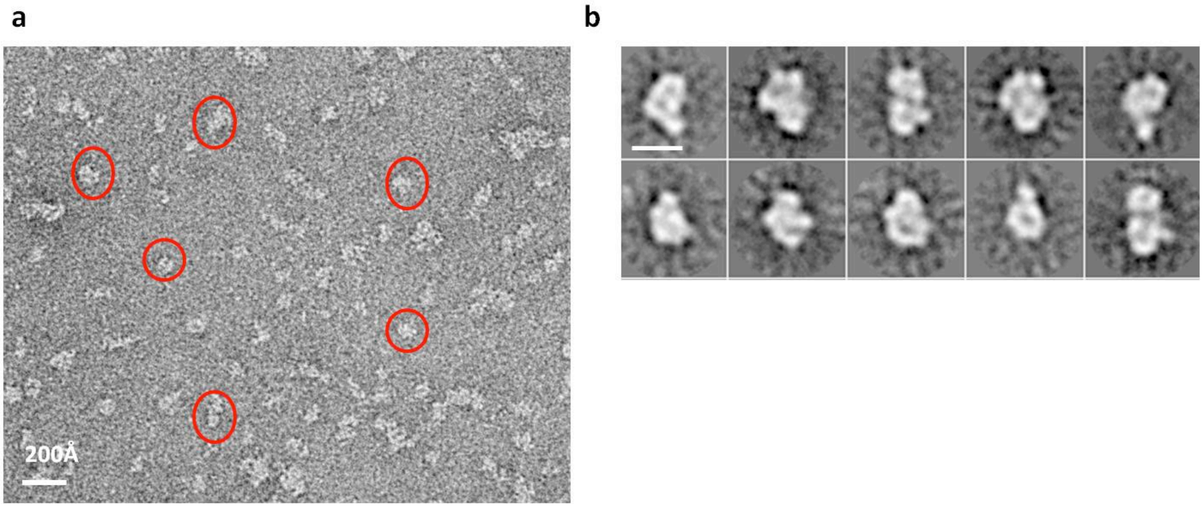

Supplementary Figure 5. Negative staining EM analysis of the yeast Not2-Not5 heterodimer. a) An example of a negatively stained image of the Not2-Not5 dimer with some selected individual particles (red circles). **b)** Example of the representative reference-free 2D classes. Bar in b) = 100 Å.

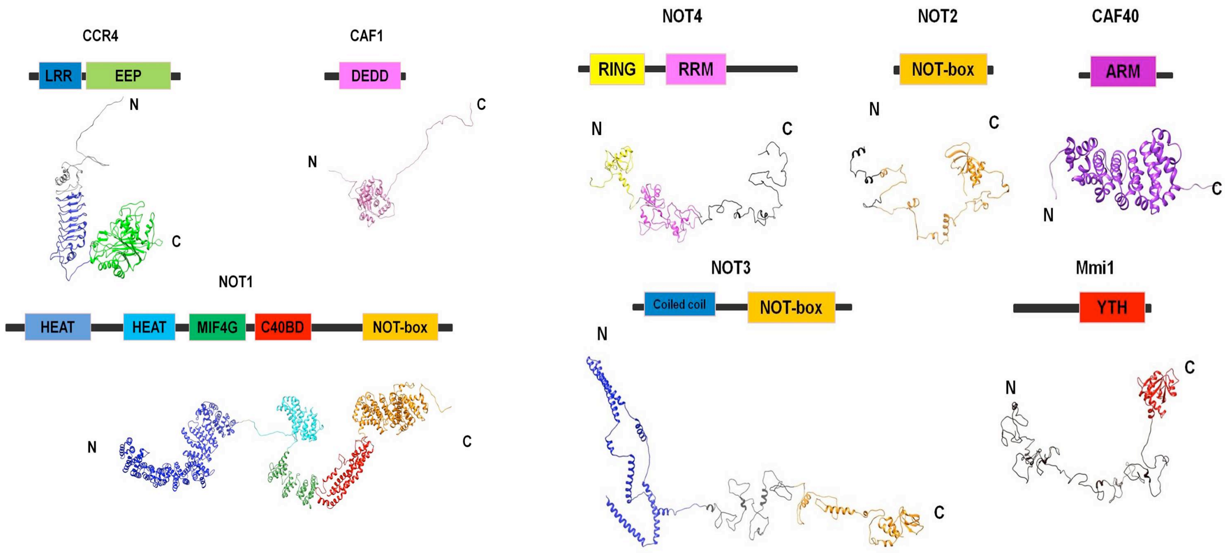

Supplementary Figure 6. Predicted homology structures of the subunits of the *S. pombe* CCR4-NOT complex. Schemes above each predicted structure represent domain organization of each subunit.

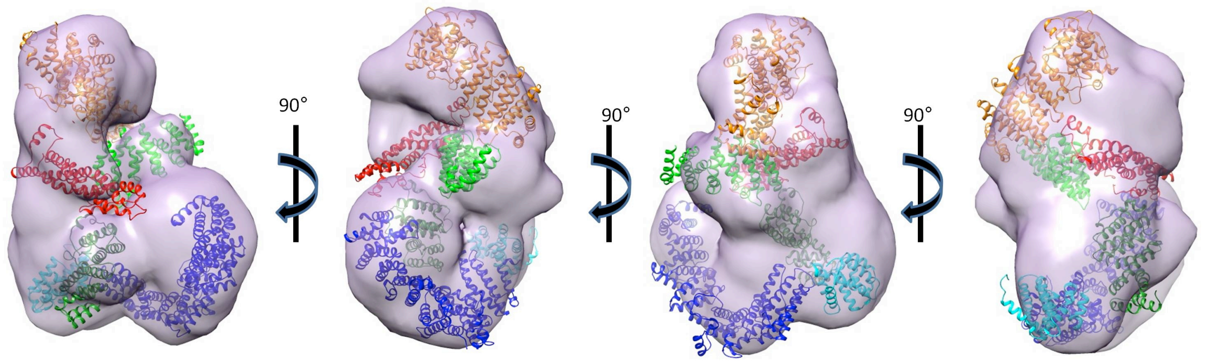

Supplementary Figure 7. Docking of the homology structure for Not1 into the cryo-EM 3D reconstruction of the CCR4-NOT complex. Not1 is a scaffold of the CCR4-NOT complex, spans the whole volume and provides a platform for protein-protein interactions. Colors show domain organization: NOT-box (orange), C40BD (red), MIF4G (green), HEAT (light and dark blue).

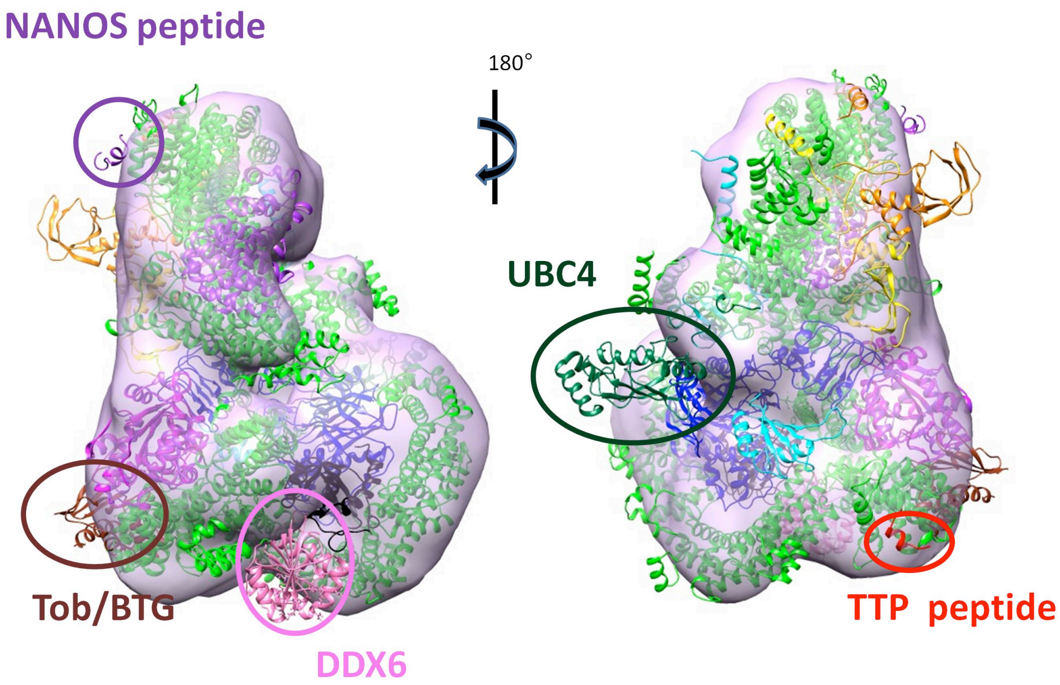

Supplementary Figure 8. Pseudo-atomic model of the CCR4-NOT complex with the interacting partners. Crystal structures of the known interacting partners (Nanos, Tob/BTG, DDX6, TTP and Ubc4) and the fragments of the CCR4-NOT complex (4CQO, 2D5R, 4CT4, 4J8S and 5AIE) were superimposed on the pseudo-atomic model showing their binding sites in the context of the entire complex.

–

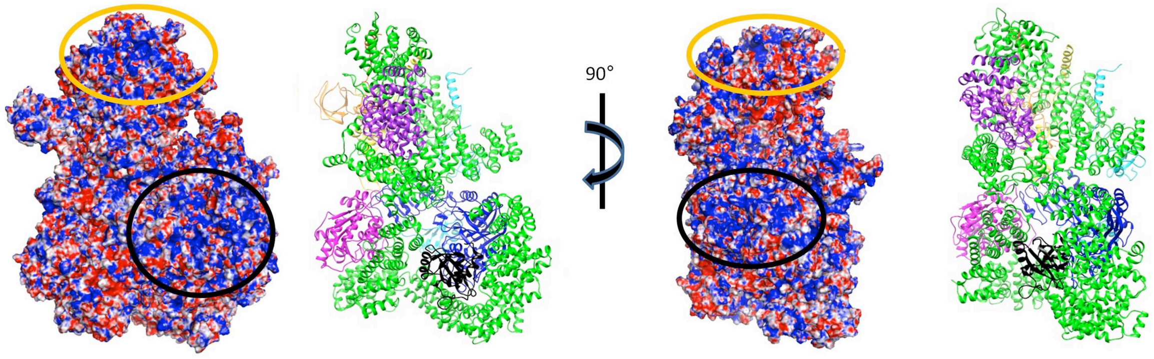

Supplementary Figure 9. Electrostatic potential of the yeast CCR4-NOT complex. Different views of the CCR4-NOT complex, electrostatic potential (blue and red represent positive and negative charges, respectively) and views of the corresponding pseudo-atomic model. Subunits: Ccr4 (dark blue), Caf1 (pink), Not1 (green), Caf40 (purple), Not2 (red), Not3 (orange), Not4 (blue), Mmi1 (black). Black and orange circles indicate the Mmi1- and Not-module related patches of positive charge (see main text).

Supplementary Table 1. List of *Schizosaccharomyces pombe* strains used in this study

Name of strain	Genotype of strain
NOT2-TAP	h+ NOT2-TAP::kan
NOT2-TAP Mmi1-ProteinA	h- NOT2-TAP::kan Mmi1-ProteinA::natMX6
NOT2-TAP NOT3/5GFP	h+ NOT2-TAP::kan NOT3/5GFP::natMX6
NOT2-TAP NOT4GFP	h+ NOT2-TAP::kan NOT4GFP::natMX6
NOT2TAP POP2GFP	h+NOT2TAP::kan POP2GFP::natMX6
NOT2TAP NOT1GFP	h- NOT2TAP::kan NOT1GFP::natMX6
NOT2TAP CAF40GFP	h+ NOT2-TAP::kan CAF40GFP::natMX6
NOT2TAP CCR4GFP	h+ NOT2-TAP::kan CCR4GFP::natMX6

Supplementary Table 2. Number of single particles selected for each 3D reconstruction

**CCR4-NOT complex characteristic**	**Number of particles**
NOT2-TAP, Negative staining EM	10,303
NOT2-TAP, Mmi1-ProteinA	10,331
NOT2-TAP, NOT1-GFP	21,715
NOT2-TAP, POP2-GFP	14,700
NOT2-TAP, CCR4-GFP	15,690
NOT2-TAP, NOT3-GFP	12,846
NOT2-TAP, CAF40-GFP	13,280
NOT2-TAP, NOT4-GFP	17,560
NOT2-TAP-RNA-nanoGold labeling	8,500
NOT2-TAP, cryo-EM	60,480

Supplementary Table 3. Description of structures of CCR4-NOT components used in hybrid modeling with the PyRy3D program.

**CCR4**
1-90 disorder<br>91-150 model, very low accuracy (possible disorder)<br>151-280 model, high accuracy<br>281-317 disorder (linker)<br>318-676 model, high accuracy (low accuracy: 528-548, 583-595)<br>677-690 disorder (linker)
**RCD1**
1-10 disorder<br>11-275 model, very high accuracy<br>276-283 disorder
**CAF1**
1-22 disorder<br>23-273 model, very high accuracy (X-ray structure)<br>274-335 disorder
**NOT1**
1-35 disorder<br>36-653 model, low accuracy<br>654-655 disorder<br>656-815 model, very high accuracy<br>816-840 disorder<br>841-1090 model, very high accuracy<br>1091-1096 disorder<br>1097-1328 model, high accuracy<br>1329-1567 model, low accuracy<br>1568-1582 disorder<br>1583-2072 model, very high accuracy<br>2073-2100 disorder
**NOT2**
1-127 disorder<br>128-306 model, high accuracy
**NOT3**
1-490 disorder<br>491-634 model, high accuracy<br>635-640 disorder
**NOT4**
1-15 disorder<br>16-76 model, high accuracy<br>77-112 disorder<br>113-200 model, high accuracy<br>201-489 disorder
**Mmi1**
1-349 disorder<br>350-487 model, high accuracy
